# Down-regulation of human-specific lncRNA TMEM9B-AS1 in skeletal muscle of people with type 2 diabetes affects ribosomal biogenesis

**DOI:** 10.1126/sciadv.ads4371

**Published:** 2025-07-09

**Authors:** Ilke Sen, Jonathon A. B. Smith, Elena Caria, Iurii Orlov, Mladen Savikj, Aidan J. Brady, Kristian Lian, Stian Ellefsen, Juleen R. Zierath, Anna Krook

**Affiliations:** ^1^ Department of Physiology and Pharmacology, Karolinska Institutet, Stockholm 171 77, Sweden.; ^2^ Faculty of Medicine, Mondor Institute for Biomedical Research, INSERM U955, Université Paris Est Créteil, 94010 Créteil, France.; ^3^ Department of Molecular Medicine and Surgery, Karolinska Institutet, Stockholm 171 77, Sweden.; ^4^ Section for Health and Exercise Physiology, Department of Public Health and Sport Sciences, Inland Norway University of Applied Sciences, Lillehammer, Norway.

## Abstract

Long noncoding RNAs (lncRNAs) are important regulators of skeletal muscle physiology, with altered expression noted in several human diseases including type 2 diabetes. We report that TMEM9B-AS1, a previously uncharacterized lncRNA, is down-regulated in skeletal muscle of men with type 2 diabetes and skeletal muscle from individuals with sarcopenia. Silencing of TMEM9B-AS1 in primary human myotubes attenuated protein synthesis, concomitant with reduced phosphorylation of ribosomal protein S6. Moreover, we show that TMEM9B-AS1 plays a pivotal role in regulation of ribosomal biogenesis by facilitating messenger RNA stabilization of the transcription factor MYC through direct physical interaction with the RNA binding protein, insulin-like growth factor 2 mRNA binding protein 1 (IGF2BP1). Disrupted ribosomal biogenesis resulting from TMEM9B-AS1 silencing leads to decreased expression of muscle contractile and structural proteins important for maintenance of skeletal muscle mass and function. Collectively, our data reveal a role of TMEM9B-AS1 in skeletal muscle loss associated with metabolic disorders.

## Teaser

TMEM9B-AS1, down-regulated in type 2 diabetes, regulates ribosomal biogenesis in muscle by promoting IGF2BP1-MYC mRNA interaction.

## INTRODUCTION

Long noncoding RNAs (lncRNAs) make up a considerable part of the genome and encompass a diverse class of RNA molecules that are not translated into proteins (*1*). As important regulators of cell and tissue function (*2*), lncRNAs have been implicated in the development of metabolic diseases. Type 2 diabetes is a metabolic disease exacting an ever-increasing toll on public health. The etiology of type 2 diabetes is complex, involving multiple tissues resulting in impaired glucose homeostasis. While several lncRNAs have been shown to regulate glucose and lipid metabolism in liver and adipose tissue (*3*, *4*), as well as functional properties of pancreatic islets (*3*), effects in skeletal muscle are not clearly understood. Skeletal muscle comprises ~50% of body mass, and as the primary site for insulin-stimulated glucose disposal, impaired insulin action in this organ often presents early in type 2 diabetes pathogenesis (*5*). Furthermore, type 2 diabetes is associated with a decline in muscle mass (*6*), which can be exacerbated by GLP-1 receptor agonists prescribed as weight loss therapy (*7*–*9*).

Functional roles attributed to lncRNAs include the regulation of transcriptional and translational processes (*4*). Cytoplasmic lncRNAs interact with RNA binding proteins to affect mRNA stability of specific genes and thereby coordinate protein synthesis/translation (*10*). Protein synthesis rates result from the efficiency and capacity of the translation machinery. As total ribosomal content dictates translational capacity, ribosomal biogenesis is important for the maintenance and hypertrophy of skeletal muscle mass (*11*, *12*). Ribosome biogenesis is modulated by several signaling pathways, with ncRNAs emerging as notable regulators of this process (*13*). Although the precise mechanisms remain unclear, the phosphatidylinositol 3-kinase/mammalian target of rapamycin (mTOR, also known as mechanistic target of rapamycin)/p70S6K1 network and the transcription factor MYC appear necessary for ribosomal biogenesis in skeletal muscle (*14*, *15*).

In a search for lncRNAs associated with insulin resistance, we identified lncRNA TMEM9B-AS1 expression is down-regulated in skeletal muscle of men with type 2 diabetes. Contextualizing the role of this differentially expressed lncRNA in metabolic processes, we provide evidence that TMEM9B-AS1 is required for protein synthesis and regulates ribosomal biogenesis by facilitating the stability of MYC mRNA through physical interaction with the RNA binding protein insulin-like growth factor 2 mRNA binding protein 1 (IGF2BP1). This interaction is essential for ribosomal biogenesis and protein synthesis in myotubes and for the expression of muscle contractile and structural proteins. Thus, maintaining sufficient levels of TMEM9B-AS1 could be a potential target to improve metabolic health and physical performance by ameliorating detriments in skeletal muscle mass and function.

## RESULTS

### TMEM9B-AS1 is down-regulated in skeletal muscle of men with type 2 diabetes and is localized to the cytoplasm in primary human myotubes

**Fig. 1.**
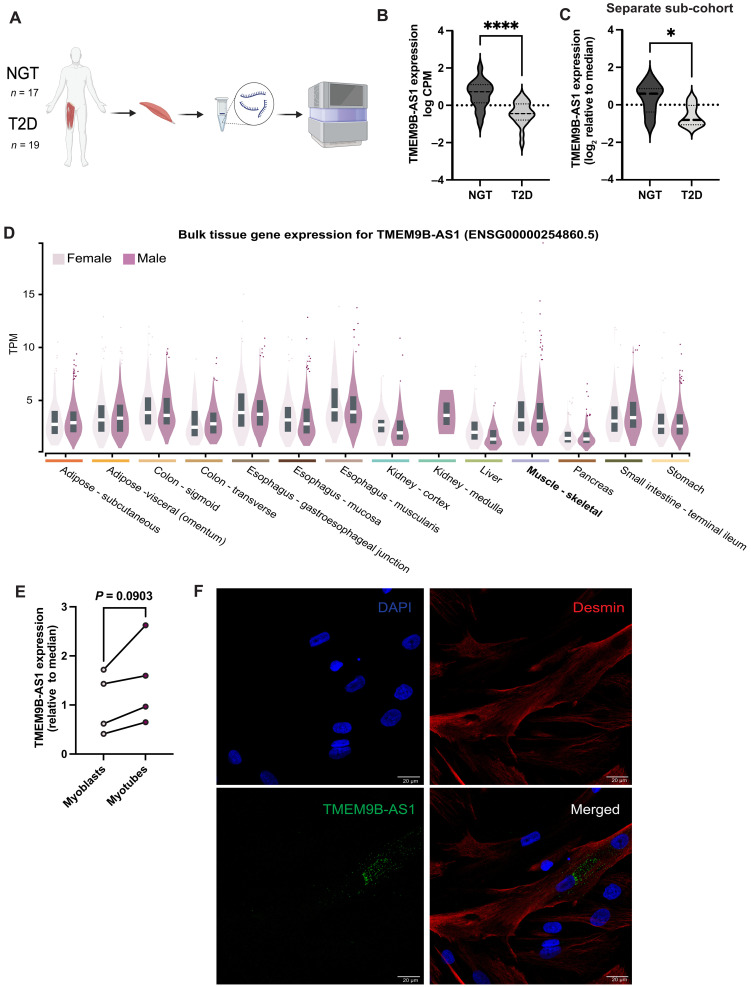
TMEM9B-AS1 is down-regulated in skeletal muscle from men with type 2 diabetes and expressed in the cytoplasm in human myotubes. (**A**) Schematic representation of the study design. Created in BioRender. I. Sen (2025) https://BioRender.com/4spzvf7. (**B**) RNA sequencing (RNA-seq) data showing a decrease in TMEM9B-AS1 expression levels in skeletal muscle of men with type 2 diabetes (*n* = 19) compared to men with normal glucose tolerance (*n* = 17). For the statistical analysis, an unpaired *t* test was performed; ****P < 0.0001. (**C**) Gene expression analysis with TaqMan assays in a separate sub-cohort of people with type 2 diabetes (*n* = 6) and matched normal glucose–tolerant people (*n* = 7). For the statistical analysis, an unpaired *t* test was performed; *P < 0.05. Data were normalized to the housekeeping genes B2M and TBP. (**D**) Bulk tissue gene expression levels of TMEM9B-AS1 in several metabolic tissues from both women and men. Data were extracted from the GTEX database (*59*). (**E**) Gene expression analysis with TaqMan assays showing the expression of TMEM9B-AS1 in human skeletal muscle myoblasts and differentiated myotubes. Data were normalized to the housekeeping genes B2M and TBP, (*n* = 4). For the statistical analysis, a two-tailed, paired *t* test was performed. (**F**) Confocal images showing the localization of TMEM9B-AS1 by RNA FISH combined with immunofluorescence. Desmin was used as a structural protein marker for human myotubes, and 4′,6-diamidino-2-phenylindole (DAPI) was used to stain the nucleus. Scale bar, 20 μm. Experiments were performed using biological replicates.

Differentially expressed transcripts were determined from skeletal muscle biopsies obtained from a previously published cohort of age and BMI-matched men, 19 with type 2 diabetes and 17 with normal glucose tolerance (Fig. 1A) (*16*). TMEM9B-AS1, a human-specific antisense lncRNA, was down-regulated in skeletal muscle of individuals with type 2 diabetes (Fig. 1B). Reduced TMEM9B-AS1 expression was validated in a subgroup of individuals with type 2 diabetes and normal glucose tolerance from a separate cohort (Fig. 1C) (*17*). Bulk expression of TMEM9B-AS1 in different human tissues from both women and men was probed in the GTEX database, revealing broad tissue distribution of TMEM9B-AS1 expression (Fig. 1D). Since several of the participants with type 2 diabetes were treated with metformin or statins, we determined whether these drugs would directly alter TMEM9B-AS1 expression in human muscle cultures. TMEM9B-AS1 levels were unaltered following a 3-hour exposure of myotubes to metformin (5 μM, 200 μM, or 2 mM) or simvastatin (5 μM) (fig. S1A). Thus, reduced TMEM9B-AS1 levels appear to be a feature of type 2 diabetes rather than a response to metformin or simvastatin treatment.

Skeletal muscle is a complex heterogeneous tissue that consists of multinucleated muscle fibers, endothelial cells, satellite cells (muscle stem cells), immune cells, and other mononuclear cells (*18*). To investigate whether TMEM9B-AS1 is expressed in muscle cells, we determined TMEM9B-AS1 expression using quantitative polymerase chain reaction (qPCR) in primary human skeletal muscle cells. TMEM9B-AS1 was expressed in human myoblasts as well as differentiated human myotubes (Fig. 1E). To determine the subcellular localization of TMEM9B-AS1, we used RNA–fluorescence in situ hybridization (FISH) combined with immunocytochemistry (Fig. 1F). Human myotubes were labeled using a specific antibody against desmin, a muscle-specific protein important for myotube structure, and a specific probe to detect TMEM9B-AS1 RNA. We observed that TMEM9B-AS1 is localized in the cytoplasm (Fig. 1F).

### Reduction in TMEM9B-AS1 affects protein synthesis through mTOR and ERK signaling

**Fig. 2.**
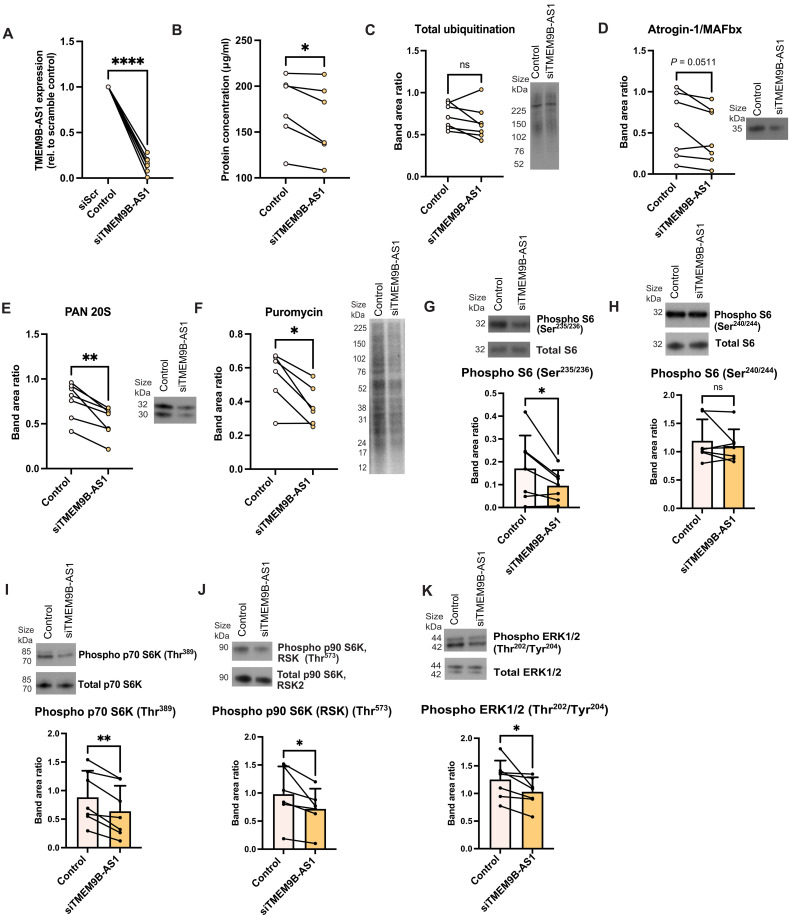
Reduction in TMEM9B-AS1 affects protein synthesis through mTOR and ERK signaling. (**A**) qPCR (TaqMan assay) results showing silencing efficiency of TMEM9B-AS1 in human myotubes in seven different donors. Data were normalized to the housekeeping genes B2M and TBP. For the statistical analysis, a one-sample *t* test was performed; ****P < 0.0001. (**B**) BCA results showing a decrease in total protein levels upon TMEM9B-AS1 silencing, *n* = 6. Western blot results of (**C**) Total ubiquitination (*n* = 7), (**D**) Atrogin/MAFbx (*n* = 7), and (**E**) alpha subunits of the 20S proteasome (Pan 20Sα) (*n* = 7). (**F**) Western blot results of puromycin following an SUnSET assay, *n* = 6. Western blot results showing the level of phosphorylation of (**G**) S6 protein on Ser^235/236^ (*n* = 7), (**H**) S6 protein on Ser^240/244^ (*n* = 7), (**I**) p70 S6K on Thr^389^ (*n* = 7), (**J**) p90 S6K (RSK) on Thr^573^ (*n* = 6), and (**K**) ERK1/2 on Thr^202^/Tyr^204^ (*n* = 7) upon TMEM9B-AS1 silencing. Phosphorylation levels were normalized to total protein levels following normalization to a selected Ponceau band. For the statistical analysis, a two-tailed, paired *t* test was performed; *P < 0.05, **P < 0.01. ns, not significant. Experiments were performed using biological replicates.

To investigate the role of TMEM9B-AS1 in skeletal muscle metabolism, we silenced TMEM9B-AS1 expression with small interfering RNA (siRNA) in primary human skeletal muscle cells. TMEM9B-AS1 expression was efficiently reduced following siRNA silencing (Fig. 2A). The abundance of proteins important for muscle structure, function, and contractility, including desmin, (fig. S2A), MYH7 (slow-twitch myosin heavy chain type I; MyHC-I) (fig. S2B), and MYH1/2 (detecting fast-twitch myosin heavy chain types IIA and IIX; MyHC-IIA and MyHC-IIX) (fig. S2C) were reduced following TMEM9B-AS1 silencing, with no difference seen for myogenin (MYOG; fig. S2D). In addition, mRNA expression levels of differentiation markers PAX7, MYOD1, and MYOG were unaltered by silencing of TMEM9B-AS1 (fig. S2E).

TMEM9B-AS1 silencing did not affect glucose oxidation (fig. S3A), lipid (palmitic acid) oxidation (fig. S3B), or glucose uptake (fig. S3C) in human myotubes at baseline or following appropriate stimulation. However, decreased total protein content was a consistent finding following TMEM9B-AS1 silencing in human myotubes (Fig. 2B). Thus, we postulated that TMEM9B-AS1 may play a role in skeletal muscle protein metabolism, including protein synthesis/translation or protein degradation, which are dynamically regulated processes that orchestrate the gain or loss of muscle mass (*19*, *20*). To determine whether TMEM9B-AS1 is involved in protein degradation, we assessed effects on the ubiquitin-proteasome system, which is activated during muscle atrophy and may contribute to loss of muscle mass (*20*). Proteins undergo proteasomal degradation in the ubiquitin-proteasome system (*20*, *21*) following ubiquitination by E3 ubiquitin ligases (*20*, *22*). Upon siRNA silencing of TMEM9B-AS1, no differences were observed on the total ubiquitination of proteins (Fig. 2C) or the abundance of muscle-specific E3 ligase, atrogin-1/MAFbx (Fig. 2D). In contrast, we detected the reduction of core subunits (1, 2, 3, 5, 6, and 7) of proteasome 20S alpha (Fig. 2E) that are typically elevated in catabolic conditions modulating muscle loss (*22*). Together, these findings indicate that the reduction in total protein content was not attributable to protein degradation, thus suggesting the potential regulation of protein synthesis by TMEM9B-AS1.

To determine whether TMEM9B-AS1 plays a role in protein synthesis, we performed surface sensing of translation (SUnSET) assay, tracing puromycin incorporation into newly synthesized proteins. Subsequently, we found that TMEM9B-AS1 silencing in human myotubes resulted in a decrease in protein synthesis/translation (Fig. 2F). Lentivirus-mediated overexpression of TMEM9B-AS1 in human myotubes (fig. S4A) increased total RNA content post-transduction (fig. S4B) and enhanced translational capacity (fig. S4, A and B) (*12*). Increased TMEM9B-AS1 expression also enhanced protein synthesis, as indicated by increased puromycin incorporation in an SUnSET assay (fig. S4C). To understand how TMEM9B-AS1 regulates protein synthesis/translation, we first focused on the mTORC1 pathway as a master regulator of protein synthesis/translation (*20*, *23*). No changes were noted on mTOR phosphorylation (fig. S4D) or on the downstream target 4E-BP1 (fig. S4E) following TMEM9B-AS1 silencing, suggesting that the mTOR signaling pathway is selectively responsive to changes in TMEM9B-AS1. However, assessing ribosomal protein S6, representing a separate signaling branch downstream of mTORC1 (*24*), we observed that phosphorylation of ribosomal protein S6 on Ser^235/236^ (Fig. 2G), but not Ser^240/244^ (Fig. 2H) was decreased by silencing of TMEM9B-AS1. Consistent with this, overexpression of TMEM9B-AS1 increased phosphorylation of ribosomal protein S6 on Ser^235/236^ (fig. S4F), but not Ser^240/244^ (fig. S4G). While phosphorylation of S6 protein on the Ser^235/236^ site is regulated by two S6Ks: p70 S6K (downstream of mTORC1) and p90 S6Ks [ribosomal protein S6 kinases (RSKs); downstream of extracellular signal–regulated kinase (ERK) signaling], the Ser^240/244^ phosphorylation site is targeted specifically by p70 S6K (*25*). These data suggested that two of the upstream S6-kinases, p70 and p90 could be involved in TMEM9B-AS1–sensitive phospho-regulation of S6 protein. Next, we determined whether loss of TMEM9B-AS1 affected the phosphorylation of p70 and p90 S6-kinases and noted that phosphorylation of p70S6K Thr^389^ (Fig. 2I) and p90S6K Thr^573^ (Fig. 2J) was reduced following silencing of TMEM9B-AS1. p90 S6 kinase is regulated by ERK 1/2, and silencing of TMEM9B-AS1 in human myotubes also decreased ERK1/2 phosphorylation on Thr^202^/Tyr^204^ (Fig. 2K). Together, our data suggest that TMEM9B-AS1 regulates protein synthesis/translation through ERK and partly through mTOR pathways and the phosphorylation of the downstream S6 protein, specifically by modulating the Ser^235/236^ phosphorylation site that is targeted by p70 and p90 S6Ks. Phosphorylation of S6 protein is necessary for translation of ribosomal proteins required for ribosomal biogenesis (*26*, *27*), which indicated that TMEM9B-AS1 may be involved in the regulation of ribosomal biogenesis.

### TMEM9B-AS1 regulates ribosomal biogenesis

**Fig. 3.**
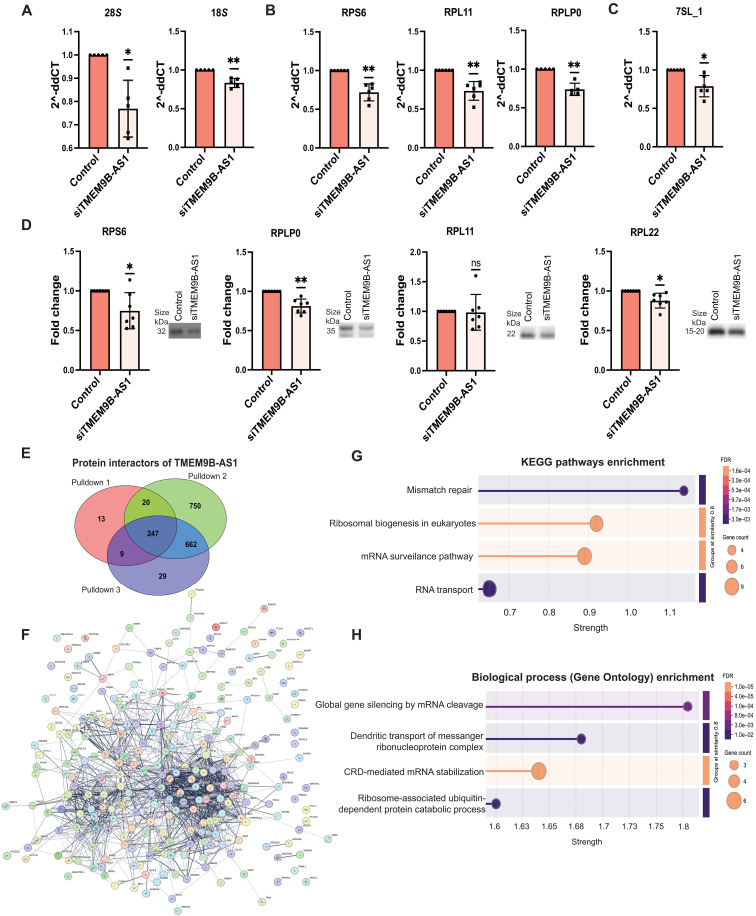
TMEM9B-AS1 regulates ribosomal biogenesis in human myotubes. qPCR (Syber green) results showing expression levels of (**A**) ribosomal RNAs, 28*S* and 18*S* (*n* = 5) (**B**) ribosomal genes, RPS6, RPL11, and RPLP0 (*n* = 6). (**C**) Ribosomal RNA, 7SL_1 (*n* = 6) upon TMEM9B-AS1 silencing. TBP and GUSB were used as housekeeping genes. For the statistical analysis, a one-sample *t* test was performed; *P < 0.05, **P < 0.01. (**D**) Western blot results of ribosomal subunit proteins RPL in human myotubes upon silencing of TMEM9B-AS1 (*n* = 7). For the statistical analysis, a one-sample *t* test was performed; *P < 0.05, **P < 0.01. (**E**) Results of the RNA pull-down experiment combined with mass spectroscopy. Venn diagrams show the number of specific binding partners of TMEM9B-AS1 in three different RNA pulldowns and their overlaps. (**F**) Protein-protein interaction networks achieved from STRING database. (**G**) KEGG pathway and (**H**) GO-term biological processes functional enrichment analysis conducted on STRING database using specific protein interactors of TMEM9B-AS1 found in all pulldowns. For both analyses, the four most enriched terms are displayed in the figures. Experiments were performed using biological replicates. Paired *t* test analyses were also performed on the raw band area ratios of the data presented in (A) to (D), which can be found in fig. S9.

De novo synthesis of ribosomes requires coordinated synthesis of equimolar quantities of four types of ribosomal RNA subunits and more than 80 ribosomal proteins. This, in turn, involves all three polymerases, Pol I, Pol II, and Pol III, each of which interacts with the master transcription factor MYC, serving as a direct regulator of ribosome biogenesis (*24*). To determine the involvement of TMEM9B-AS1 in ribosomal biogenesis, we measured expression of several ribosomal genes/RNAs that depend on Pol I (Fig. 3A), Pol II (Fig. 3B), and Pol III (Fig. 3C) following siRNA silencing of TMEM9B-AS1 in human myotubes. The expression of a subset of ribosomal genes/RNAs—18*S*, 28*S*, RPS6, RPL11, RPLP0 and 7SL_1, collectively mediated by all three polymerases—were markedly decreased upon reduction of TMEM9B-AS1 (Fig. 3, A to C). We also examined the expression of several ribosomal subunit proteins and observed a significant decrease in RPS6, RPLP0, and RPL22 expression, while RPL11 remained unchanged (Fig. 3D). However, TMEM9B-AS1 overexpression had no effect on the abundance of ribosomal genes/RNAs 48 and 96 hours post-transduction (fig. S5, A to C) or on ribosomal subunit proteins RPS6, RPLP0, RPL11, and RPL22 96 hours post-transduction (fig. S5D).

To probe whether TMEM9B-AS1 directly interacts with MYC, we performed an RNA pull-down assay using TMEM9B-AS1 as bait, followed by Western blot analysis for MYC. We did not observe a direct interaction between TMEM9B-AS1 and MYC. Therefore, to understand how TMEM9B-AS1 regulates ribosomal biogenesis, we took an unbiased approach to assess potential protein interactions with TMEM9B-AS1 using an RNA pull-down experiment followed by mass spectroscopy. A list of protein interactors of TMEM9B-AS1 was generated by considering the overlapping proteins as specific interactors in three individual pull-downs (Fig. 3E and table S1). Using this list, we probed the STRING database for potential protein-protein interaction networks (Fig. 3F) and performed functional enrichment analysis using KEGG pathway enrichment (Fig. 3G) and (Gene Ontology) GO term biological processes (Fig. 3H). We observed that protein interactors of TMEM9B-AS1 were enriched in pathways related to ribosomal biogenesis (Fig. 3G). Functional enrichment analysis for biological processes revealed that protein interactors of TMEM9B-AS1 were also enriched in coding region instability determinant (CRD)–mediated mRNA stabilization processes derived by RNA binding proteins. CRD is a 249-nucleotide region on MYC mRNA that is required for its stabilization (*28*). These data suggest that TMEM9B-AS1 may play a role in regulating MYC mRNA stabilization, which in turn could influence ribosomal biogenesis. We then analyzed publicly available ribosome profiling data from various tissues and cell types and identified the presence of TMEM9B-AS1 (*29*), supporting our hypothesis that TMEM9B-AS1 is physically associated with ribosomes.

### TMEM9B-AS1 regulates MYC mRNA stabilization by physically interacting with the RNA binding proteins, IGF2BPs

**Fig. 4.**
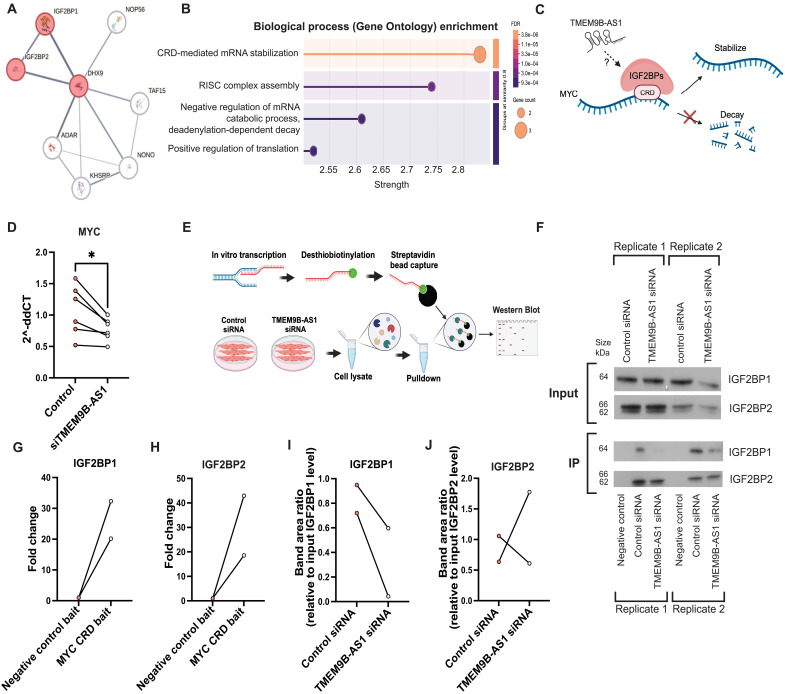
TMEM9B-AS1 regulates MYC mRNA stability through physical interaction with RNA-binding IGF2BP1in human myotubes. (**A**) Unique protein interactors of TMEM9B-AS1, determined by using the overlapping proteins in three individual TMEM9B-AS1 RNA pulldowns and TMEM9B-AS1–binding proteins according to the CLIP-SEQ data found in publicly available ENCORI database. (**B**) Functional enrichment analysis for the biological processes on STRING database using unique protein interactors of TMEM9B-AS1 achieved by the comparison of specific interactors of TMEM9B-AS1 from RNA pull-down data and CLIP-SEQ data from ENCORI database. The figure displays the top four most enriched terms. (**C**) Schematic representation of interaction between MYC mRNA and RNA binding proteins, IGF2BPs via the CRD region on MYC mRNA, which facilitates the stabilization of MYC mRNA. Created in BioRender. I. Sen (2025) https://BioRender.com/a43u001. (**D**) mRNA levels of MYC upon TMEM9B-AS1 silencing. qPCR data normalized to the housekeeping gene, TBP (*n* = 6). For the statistical analysis, a two-tailed paired *t* test was performed; *P < 0.05. (**E**) Schematic representation of RNA pull-down experiments to determine the binding efficiency of RNA binding proteins IGF2BP1 and IGF2BP2 to CRD region on MYC mRNA in the presence or absence of TMEM9B-AS1. Created in BioRender. I. Sen (2025) https://BioRender.com/k2yxvbk. (**F**) Western blot results for IGF2BP1 and IGF2BP2 upon RNA pulldowns. Negative control RNA was used to determine the RNA pull-down specificity over MYC CRD. RNA pull-down specificity of (**G**) IGF2BP1 and (**H**) IGF2BP2. RNA binding efficiency of (**I**) IGF2BP1 and (**J**) IGF2BP2 upon two RNA pulldowns in control and TMEM9B-AS1 silencing conditions. Data were normalized to the individual input levels of IGF2BP1 and IGF2BP2. Experiments were performed using biological replicates.

To further determine specific interactors of TMEM9B-AS1, we compared the list of protein interactors in the RNA pull-down–mass spectrometry data with TMEM9B-AS1–binding proteins identified in publicly available CLIP-SEQ data in the ENCORI/starBase database (*30*). From this confirmation, we generated a short list of unique interactors of TMEM9B-AS1 (Fig. 4A). Using this short list of proteins, we performed functional enrichment analysis for the biological processes and found that the top GO term was CRD-mediated mRNA stabilization, mostly derived from the RNA binding proteins, IGF2BP1 and IGF2BP2 (Fig. 4, A and B). IGF2BPs are highly conserved RNA binding proteins that play a role in regulating RNA processing at different stages, including localization, translation, and stability (*31*, *32*).

IGF2BP1 stabilizes MYC mRNA by associating with its CRD region in cell culture models (*24*, *28*, *32*) (illustrated in Fig. 4C), and we hypothesized that TMEM9B-AS1 could play a role in regulating this process. Accordingly, MYC mRNA levels were reduced in human myotubes following TMEM9B-AS1 silencing (Fig. 4D), suggesting that TMEM9B-AS1 could regulate MYC mRNA stability via a physical interaction with RNA binding proteins, IGF2BPs. To probe this possibility, we performed an RNA pull-down experiment using the MYC-CRD region as bait to determine whether TMEM9B-AS1 is required for the interaction between IGF2BP1 or IGF2BP2 and the CRD region on MYC mRNA (illustrated in Fig. 4E). Pull-down experiments were performed using cell lysates from human myotubes in the presence or absence of TMEM9B-AS1 silencing. The ability of MYC-CRD region to pull down IGF2BP1 and/or IGF2BP2 was assessed using Western blot analysis (Fig. 4, F to J). Pull-down efficiencies and specificities for IGF2BP1 (Fig. 4G) and IGF2BP2 (Fig. 4H) were similar. We observed a consistent decrease in the pull-down efficiency of the MYC-CRD region in cells lacking TMEM9B-AS1 for IGF2BP1 (Fig. 4, F and I), but not IGF2BP2 (Fig. 4, F and J).

IGF2BP1 and IGF2BP2 are expressed in human myotubes and siRNA silencing of TMEM9B-AS1 decreases IGF2BP1 protein levels but not IGF2BP2 (fig. S6, A and B), which indicates that TMEM9B-AS1 could also regulate the protein stability of IGF2BP1. We further investigated whether translation of MYC is altered in human myotubes in the absence of either TMEM9B-AS1, IGF2BP1, or IGF2BP2, but did not observe any changes in MYC protein levels (fig. S6, C and D).

### IGF2BP1, but not IGF2BP2, regulates gene expression important for ribosomal biogenesis

**Fig. 5.**
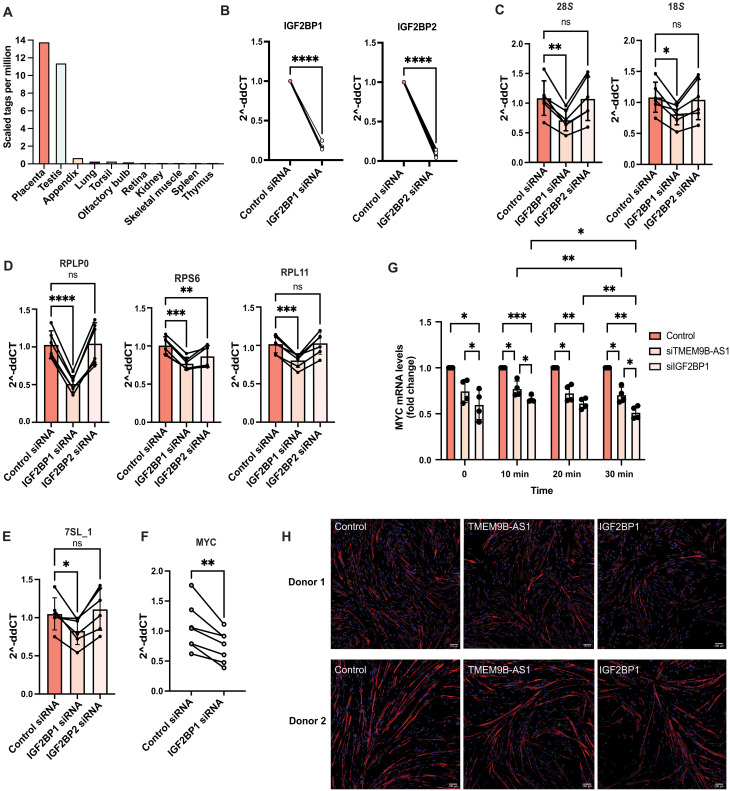
IGF2BP1 not IGF2BP2 regulates ribosomal biogenesis in human skeletal muscle cells. (**A**) Expression of IGF2BP1 in different human tissues from FANTOM 5 data. (**B**) qPCR (TaqMan assay) results showing the siRNA silencing efficiencies of IGF2BP1 and IGF2BP2. For the statistical analysis, a one-sample *t* test was performed; ****P < 0.0001. qPCR (Syber green) results showing the expression levels of (**C**) ribosomal RNAs, 28*S* and 18*S*; (**D**) ribosomal genes, RPLP0, RPS6, and RPL11; and (**E**) ribosomal RNA, 7SL_1 upon IGF2BP1 and IGF2BP2 silencing. TBP and GUSB were used as housekeeping genes (*n* = 6). For the statistical analysis, a one-way ANOVA test with post hoc Dunnett’s test for comparisons to control was performed; *P < 0.05, **P < 0.01, ***P < 0.001. (**F**) qPCR (TaqMan assay) results showing mRNA levels of MYC upon IGF2BP1 silencing. TBP, PPIA, and HPRT were used as housekeeping genes (*n* = 7). For the statistical analysis, a two-tailed, paired *t* test was performed; **P < 0.01. (**G**) Results of the time course mRNA stability experiment of human myotubes treated with actinomycin D for blocking transcription upon siRNA silencing of TMEM9B-AS1 and IGF2BP1. qPCR (TaqMan assay) data normalized to the housekeeping gene, TBP (*n* = 4). For the statistical analysis, a two-way ANOVA test, mixed-effect analysis was performed. *P* value for silencing effect is 0.0002 and for time effect is 0.29. Tukey post hoc test was applied for multiple comparisons; *P < 0.05, **P < 0.01, ***P < 0.001. (**H**) Immunocytochemistry images showing Desmin protein levels (shown as red) in differentiated human myotubes in two different donors: donor 1 and donor 2. DAPI was used for the staining of the nuclei (shown as blue). Scale bar, 100 μm. Experiments were performed using biological replicates.

We used the FANTOM5 database to confirm the pattern and level of IGF2BP1 gene expression. Although transcript abundance is low, IGF2BP1 is expressed in several adult tissues including human skeletal muscle (Fig. 5A). We explored this finding further by dissecting data from the GTEX Portal (*33*) for single tissue/cell expression of IGF2BP1, TMEM9B-AS1, and MYC, which confirmed expression in skeletal muscle tissue, mostly within myocytes (fig. S7, A and C). We validated this finding using another platform for single cell/nuclei data, CZ CELLxGENE Discover (*34*) and confirmed that IGF2BP1, TMEM9B-AS1, and MYC are expressed in skeletal muscle fibers and that TMEM9B-AS1 and IGF2BP1 have a similar expression pattern (fig. S7D). Moreover, we observed a higher expression level of IGF2BP1 in human myotubes compared to bulk skeletal muscle tissue from humans or rodents, as well as cultured rodent skeletal muscle cells (fig. S7E), suggesting possible species-specific roles for IGF2BP1 in human skeletal muscle.

To further explore whether IGF2BPs are involved in the regulation of ribosomal biogenesis in human myotubes, we determined whether siRNA silencing of IGF2BP1 or IGF2BP2 would phenocopy the TMEM9B-AS1–mediated reduction of RNAs/genes important for ribosomal biogenesis (Fig. 3, B to D). Both IGF2BPs were efficiently silenced (Fig. 5B). However, the expression levels of ribosomal RNAs/genes dependent on Pol I (Fig. 5C), Pol II (Fig. 5D), and Pol III (Fig. 5E), were only decreased following silencing of IGF2BP1, apart from RPS6. These data are compatible with a model whereby TMEM9B-AS1 physically interacts with IGF2BP1, which in turn regulates mRNA stability of MYC by mediating the interaction of the MYC-CRD region and the RNA binding protein IGF2BP1. IGF2BP1 regulates mRNA stability of MYC (*32*, *35*) in cell lines. Consistent with this, siRNA-mediated silencing of IGF2BP1 reduced MYC mRNA (Fig. 5F). MYC mRNA is unstable with a short (15 to 30 min) half-life in human cells (*36*, *37*). Using this time window, we tested whether mRNA stability of MYC is affected by the silencing of TMEM9B-AS1 and IGF2BP1 upon blocking transcription using actinomycin D treatment. We observed a decline in MYC mRNA levels upon TMEM9B-AS1 or IGF2BP1 silencing between 10 and 30 min after the treatment (Fig. 5G). In addition, in line with our previous findings (S2A), we observed altered myotube morphology following the silencing of TMEM9B-AS1 or IGF2BP1, as assessed by desmin immunofluorescence staining (Fig. 5H).

**Fig. 6.**
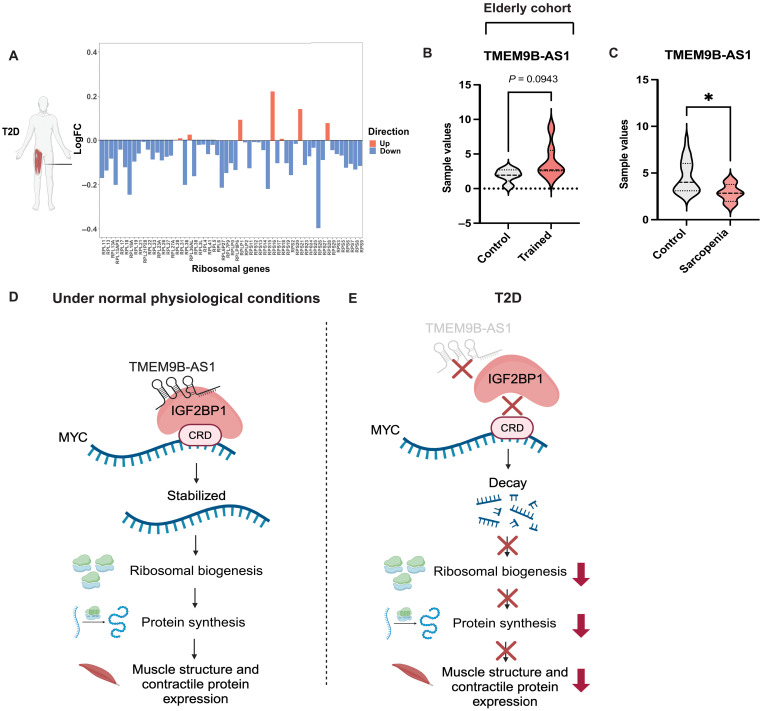
Supporting evidence for the regulation of ribosomal biogenesis and muscle mass by TMEM9B-AS1 in several human cohorts and proposed model. (**A**) Expression levels of ribosomal genes in the skeletal muscle of individuals with type 2 diabetes compared with individuals with normal glucose tolerance. Data are extracted from the original RNA-seq data. (**B**) The expression levels of TMEM9B-AS1 in GSE165630 dataset, derived from a clinical cohort following resistance or combined training (*n* = 5 for control group and *n* = 7 for trained group). Welch two-tailed *t* test was used for the statistical analysis. (**C**) TMEM9B-AS1 levels in people with sarcopenia compared to age-matched individuals without sarcopenia with normal muscle mass levels. GSE111006 dataset was used, and analyses were performed using GEO2R. Sample values are extracted and plotted. Welch two-tailed *t* test was used for the statistical analysis (*n* = 32 for control group and *n* = 4 for sarcopenia group), *P < 0.05. (**D**) Under normal physiological conditions, TMEM9B-AS1 physically binds to the RNA binding protein IGF2BP1 and mediates its interaction with MYC mRNA through its CRD region. This facilitates the stability of MYC mRNA and eventually ribosomal biogenesis, as MYC is ultimately required for the expression of ribosomal RNAs/genes constituting ribosomal subunits. (**E**) In type 2 diabetes, the decrease of TMEM9B-AS1 expression levels in the skeletal muscle disrupts the interaction between IGF2BP1 and MYC CRD is disrupted, which interferes with the mRNA stabilization of MYC mRNA. The subsequent decay eventually results in the decline of ribosomal biogenesis and decreased expression of muscle contractile and structural proteins, which may lead to impairment in muscle mass maintenance and function. Created in BioRender. I. Sen (2025) https://BioRender.com/a43u001.

Our data provide evidence for a role of TMEM9B-AS1 in the regulation of ribosomal biogenesis and, in turn, protein synthesis. These processes are highly coordinated and essential for muscle mass and function, a feature that is impaired in type 2 diabetes (*6*, *38*, *39*). To validate whether reduced TMEM9B-AS1 would correspond to reduced ribosomal gene content, we extracted all ribosomal protein–coding genes present in our original RNA sequencing (RNA-seq) data and compared expression in skeletal muscle from individuals with type 2 diabetes to individuals with normal glucose tolerance. Strikingly, most ribosomal genes were down-regulated in skeletal muscle from individuals with type 2 diabetes (Fig. 6A), coincident with reduced TMEM9B-AS1. Since ribosomal biogenesis is essential for translational capacity and required for the maintenance of skeletal muscle mass (*12*, *40*, *41*), we further probed TMEM9B-AS1 expression in publicly available datasets. In older individuals predominantly performing either resistance or varying degrees of combined (i.e., resistance + endurance) exercise training, paradigms known to increase ribosomal biogenesis and skeletal muscle hypertrophy, there was a nonsignificant tendency for increased TMEM9B-AS1 versus sedentary individuals [Fig. 6B, data extracted from GSE165630 (*42*)]. However, TMEM9B-AS1 expression was unchanged in a separate cohort of young resistance trained individuals compared to pretraining levels (fig. S8A) despite marked accretion of ribosomal RNA (*43*, *44*). In contrast, sarcopenia represents a pathological condition of reduced skeletal muscle mass and analysis of a dataset including sarcopenia revealed down-regulation of skeletal muscle TMEM9B-AS1 [Fig. 6C, data extracted from GSE111006 (*45*)].

### Proposed model

On the basis of our results, we propose the following model (Fig. 6, D and E): Under normal physiological conditions, TMEM9B-AS1 physically binds to the RNA binding protein IGF2BP1. This binding facilitates the interaction between IGF2BP1 and MYC mRNA through the MYC-CRD region. This interaction, in turn, enhances the stability of MYC mRNA, which is essential for the transcription of ribosomal RNAs and genes that constitute ribosomal subunits (Fig. 6D). This process is required for the translational capacity, contributing to the expression of structure and contractile proteins in skeletal muscle. However, in the context of type 2 diabetes, and potentially other conditions associated with impaired skeletal muscle mass (e.g., sarcopenia), TMEM9B-AS1 expression is reduced. This reduction interferes with the interaction between IGF2BP1 and MYC-CRD, leading to a disruption in the mRNA stabilization process for MYC mRNA. Consequently, this disruption results in the decay of MYC mRNA and ultimately leads to a decline in ribosomal biogenesis, which impairs translational capacity, contributing to the reduced expression of structure and contractile proteins in skeletal muscle (Fig. 6E). The disrupted ribosomal biogenesis orchestrated by TMEM9B-AS1 holds physiological implications, potentially contributing toward the decrease in skeletal muscle mass often observed in individuals with type 2 diabetes. These findings open avenues for further research and therapeutic exploration in the context of type 2 diabetes and lncRNA function.

## DISCUSSION

Here, we provide a functional role for the lncRNA TMEM9B-AS1, which is reduced in skeletal muscle in people with type 2 diabetes or sarcopenia, in the regulation of ribosomal biogenesis and protein synthesis. These processes are crucial processes for the maintenance and accretion of skeletal muscle (*13*, *14*). These effects are at least in part mediated by stabilizing MYC mRNA. In addition to effects mediated by stabilizing MYC, TMEM9B-AS1 could facilitate other downstream processes via its regulation of IGF2BP1 protein stability. Moreover, our mass spectrometry data revealed several other protein interactors of TMEM9B-AS1, and further investigations are required to determine whether interaction of TMEM9B-AS1 with these proteins may be part of the mechanisms by which TMEM9B-AS1 modulates ribosomal biogenesis, protein synthesis, or other downstream effects.

In cultured human skeletal muscle cells, TMEM9B-AS1 silencing reduced protein content and translational capacity, whereas its overexpression increased RNA content and puromycin incorporation into newly synthesized peptides in myotubes. These findings indicate that TMEM9B-AS1 plays an important role in translational capacity and protein synthesis rates. Furthermore, silencing and overexpression of TMEM9B-AS1 respectively decreased and increased phosphorylation of S6 protein, a key regulator of ribosomal biogenesis. Consistently, TMEM9B-AS1 silencing reduced the expression of ribosomal genes/RNAs and multiple ribosomal subunit proteins. However, overexpression did not alter their levels, suggesting that TMEM9B-AS1 may not be the sole limiting factor for all aspects of ribosomal biogenesis in myotubes. This discrepancy could also be attributed to missed regulatory windows within the experimental timeline. Instead, we propose that physiological levels of TMEM9B-AS1 (and/or IGF2BP1) are critical for maintaining ribosomal biogenesis and protein synthesis homeostasis. The ability of TMEM9B-AS1 to regulate MYC mRNA stability likely depends on the availability of IGF2BP1. In this model, TMEM9B-AS1 is required for muscle for growth or mass maintenance, whereas insufficient TMEM9B-AS1 impairs MYC mRNA stabilization, leading to reduced ribosomal biogenesis and protein content. Thus, an adequate presence of TMEM9B-AS1 is essential for skeletal muscle to respond effectively to growth stimuli.

Total protein content is determined by the balance between protein synthesis and degradation. While TMEM9B-AS1 silencing reduced protein synthesis markers, protein degradation markers remained unchanged. Therefore, the observed decrease in total protein levels following TMEM9B-AS1 silencing is likely attributed to diminished protein synthesis rather than increased protein degradation. Although Atrogin-1/MAFbx expression exhibited a slight downward trend, this may reflect a compensatory mechanism by which cells attempt to preserve the reduced pool of synthesized protein and protect them from degradation.

The modulation of S6 phosphorylation by TMEM9B-AS1 further supports its role in ribosomal biogenesis. In mice, loss of S6 phosphorylation leads to reduced skeletal muscle mass and strength, accompanied by decreased contractile protein levels (*46*). These findings suggest that S6 phosphorylation governs ribosome function, selectively enhancing the translation of mRNAs encoding sarcomeric proteins. Therefore, in addition to effects on overall translational capacity, TMEM9B-AS1 may regulate contractile and structural muscle proteins, such as desmin, MYH7, and MYH1/2 through S6 phosphoregulation.

IGF2BPs are oncofetal proteins predominately expressed during early development and in tumorigenesis (*47*). IGF2BP2 has been implicated in the translational regulation and ribosomal biogenesis (*48*, *49*), yet the role of IGF2BP1 in these processes remains largely unexplored. Notably, genetic variants of IGF2BP2 are associated with type 2 diabetes and metabolic phenotypes (*49*, *50*). Using the Type 2 Diabetes Knowledge Portal (*51*), we identified strong associations between IGF2BP1 variants and body weight, as well as moderate associations with height, glycated hemoglobin, and type 2 diabetes. These findings provide evidence linking IGF2BP1 to metabolic regulation and disease susceptibility.

In our study, silencing TMEM9B-AS1 significantly reduced MYC mRNA abundance in primary human myotubes. However, we did not observe corresponding changes in MYC protein abundance following TMEM9B-AS1 or IGF2BP1 silencing. This contrasts with findings in HeLa and HepG2 human cell lines, where MYC protein levels decreased upon IGF2BP1 silencing (*52*). Given the highly dynamic nature of MYC translation, these discrepancies may arise from differences in experimental timing, silencing efficiency, or intrinsic variations between primary cells and transformed cell lines.

Loss of TMEM9B-AS1 is associated with reduced skeletal muscle mass, highlighting its potential role in the maintenance of skeletal muscle mass. Currently, no approved therapies directly target skeletal muscle mass, underscoring the importance of understanding the role of lncRNAs in muscle mass regulation. Investigating the role of TMEM9B-AS1 could provide novel insights into muscle preservation across the lifespan and inform therapeutic strategies for type 2 diabetes and metabolic disorders. In addition, whether reduced TMEM9B-AS1 contributes to the attenuated hypertrophic response in aging individuals remains an open question (*53*). While our analysis focuses on the role of TMEM9B-AS1 in skeletal muscle, it may exert similar effects in other tissues. Further investigation into additional protein interactors could deepen our understanding of TMEM9B-AS1’s coordination of ribosomal biogenesis and skeletal muscle integrity.

Limitations of the present study include challenges in translating results to an in vivo model. Although we could overexpress TMEM9B-AS1 in skeletal muscle of mice to assess physiological outcomes on muscle mass and function, TMEM9B-AS1 is human specific—a feature shared with many lncRNAs (*54*). Despite mouse IGF2BP1 having 95% sequence similarity with the human homolog, RNA pull-down experiments using protein lysates from C2C12 mouse skeletal muscle cells using TMEM9B-AS1 as bait did not detect any interaction with mouse IGF2BP1. We also noted that expression levels of IGF2BP1 in mouse skeletal muscle cells were markedly reduced in comparison to human skeletal muscle cells, further underscoring species differences in this regulatory pathway. Thus, the TMEM9B-AS1/IGF2BP1 interaction appears specific for the regulation of ribosomal biogenesis in human skeletal muscle. This study focuses on the role of TMEM9B-AS1 in mature muscle, and whether TMEM9B-AS1 also regulates satellite cell differentiation remains to be established. In our clinical cohort, most participants with type 2 diabetes were medicated using metformin and a substantial number were also using statins. Although no changes in TMEM9B-AS1 levels were noted following acute simvastatin or metformin treatment of human myotubes, we cannot exclude that chronic use of these drugs could lead to changes in vivo. Moreover, the clinical cohort consisted exclusively of men; therefore, future studies should aim to extend our findings to women. However, primary human skeletal muscle cells derived from both men and women were used for in vitro experiments and similar results were obtained regardless of the donor sex.

## MATERIALS AND METHODS

### Study design

This study aimed to investigate the role of TMEM9B-AS1, an lncRNA we identified as significantly down-regulated in skeletal muscle of individuals with type 2 diabetes, in the regulation of skeletal muscle metabolism. Transcriptomic data were obtained from a previously published clinical cohort (*16*). To functionally characterize this lncRNA, we conducted experiments using primary human skeletal muscle cells from donors with normal glucose tolerance. Sample sizes for cell-based experiments were determined on the basis of prior studies. Details regarding sample sizes, replicates, and statistical analyses are provided in the figure legends and the “Statistical analysis” section.

### Human cohorts

Skeletal muscle samples from three previously published studies were used (*16*, *17*, *43*). All samples were collected with approval of the regional ethics boards and following permission of the participants. The ethical approval license numbers for the human subjects and materials in this study are Dnr: 2013/647-31/3 and Dnr: 2014-170-31M.

### RNA-seq analysis and differential expression

RNA-seq data were used from our previous study (*16*) deposited in Gene Expression Omnibus. Downstream analysis was conducted using R version v4.0. Data were filtered to keep only 13,029 genes with >10 counts in at least 70% of samples from at least one group. The dataset was then normalized using Trimmed Mean of M-Values from the EdgeR package v3.32.1, and gene biotype annotation was collected from EnsDb.Hsapiens.v86 v2.99. Two samples were excluded from downstream analyses, as clustering of Manhattan distances between samples indicated that they were outliers. A linear model was fitted using limma v3.46. The linear model had one-way design as a combination of group (normal glucose tolerant or type 2 diabetes) and time point (basal, post-exercise, and rest), while subject pairing was accounted for by duplicate correlation. Voom was applied to account for RNA-seq heteroscedasticity. Obtained *P* values from contrasts of interest were adjusted by the Benjamini and Hochberg false discovery rate method.

### Growth and differentiation of primary human skeletal muscle cells

Primary cells were obtained from muscle biopsies taken from the vastus lateralis of healthy female and male volunteers (*55*). Myoblasts were allowed to proliferate in growth medium [F12/Dulbecco’s modified Eagle’s medium (DMEM, Gibco), 25 mM glucose, 20% fetal bovine serum (FBS), and 1% penicillin-streptomycin (antibiotic-antimyotic, Thermo Fisher Scientific)]. To induce differentiation, the cells were cultured in DMEM/M199 (4:1) (Gibco) supplemented with Hepes 20 mM (Invitrogen), zinc sulfate (0.03 μg/ml), vitamin B12 (1.4 μg/ml; Sigma-Aldrich), insulin (10 μg/ml; Actrapid; Novo Nordisk), apo-transferrin (100 μg/ml; BBI Solutions), 0.5% FBS, and 1% penicillin-streptomycin (anti-anti, Thermo Fisher Scientific). After 4 to 5 days, the cells were transitioned to post-fusion medium, which consisted of DMEM/M199 (4:1), Hepes, zinc sulfate, vitamin B12, and 0.5% FBS, along with 1% penicillin-streptomycin. The cells were incubated in 7.5% CO_2_, humidified incubators at 37°C, and the medium was changed every other day during growth and differentiation.

### siRNA silencing

siRNA transfection was performed using either control siRNA, Stealth Control #1 medium GC (12935300, Invitrogen) or TMEM9B-AS1 Stealth siRNA (HSS162366, Thermo Fisher Scientific), IGF2BP1 Stealth siRNA (HSS173800, Thermo Fisher Scientific), and IGF2BP2 Stealth siRNA (HSS173802, Thermo Fisher Scientific) (10 nM final concentration) 6 days after induction of differentiation. A second transfection was conducted 48 hours later, at day 8 after the induction of differentiation. Transfections were performed for 5 hours in OptiMEM reduced serum media with Lipofectamine RNAiMAX (Invitrogen). Two days after the second transfection (i.e., day 10 of differentiation), the cells were harvested or subjected to downstream experiments.

### Plasmid transformation, expansion, and purification

TMEM9B-AS1 (TMEM9B-AS1; NR_073431.1) was cloned into a modified pLenti cytomegalovirus (CMV) Puro DEST (w118-1) vector [pLenti CMV Puro DEST (w118-1) was a gift from E. Campeau and P. Kaufman; Addgene plasmid # 17452; https://addgene.org/17452/; RRID:Addgene_17452] by GENEWIZ (Azenta Life Science, Leipzig, Germany). Bacterial transformation and propagation were performed using One Shot TOP10 Chemically Competent *Escherichia coli* cells (Invitrogen, Thermo Fisher Scientific). The plasmids, including the modified pLenti CMV Puro DEST (w118-1) empty vector (EV) and the TMEM9B-AS1 overexpression (TMEM9B-AS1^Oex^) construct, were purified using endotoxin-free buffers (catalog no. 19048) and purification columns (catalog no. 10083) from Qiagen. DNA concentration and purity were then determined using spectrophotometry.

### Lentivirus production

Human embryonic kidney (HEK) 293T cells were maintained in a growth medium consisting of DMEM (Gibco, #10569) supplemented with 10% FBS and 1% penicillin-streptomycin. For transfection, the cells were seeded into poly-l-lysine–coated T-225 flasks 24 hours before transfection and cultured until approximately 90% confluence was reached. Lentiviral production was initiated by transfecting each T-225 flask with a mixture containing 69 μg of either EV or TMEM9B-AS1^Oex^ plasmid DNA, 69 μg of psPAX2 DNA, 45 μg of pMD2.G DNA, 549 μg of polyethylenimine (PEI; at a ratio of 3 μg of PEI per μg of plasmid DNA), and phosphate-buffered saline (PBS) to a final volume of 4.5 ml. The transfection mix was then added to 40.5 ml of prewarmed growth medium and incubated with HEK293T cells for 12 to 14 hours. After this period, the transfection medium was removed and replaced with fresh medium for 2 hours, followed by a switch to growth media supplemented with 5 mM sodium butyrate. Thirty hours posttransfection, viral supernatant was collected, filtered through a 0.45-μm low protein–binding filter (Millipore), and concentrated by low-speed centrifugation (3300*g* for 30 min at 4°C) using 100-kDa molecular weight cut-off Centricon Plus-70 cartridges (UFC710008). The concentrated viral supernatant was mixed with Lenti-X concentrator (Takara Bio) at a 3:1 ratio and incubated overnight at 4°C with constant rotation. The viral concentrate was then centrifuged (1500*g* for 45 min at 4°C), and the supernatant was discarded. The resulting pellet was resuspended in PBS, aliquoted, and stored at −80°C. The viral titer was determined using Lenti-X GoStix Plus (Takara Bio) after one freeze-thaw cycle to ensure experimental consistency.

### Transduction of primary skeletal myotubes

On day 5 of differentiation, myotubes were transduced with either EV or TMEM9B-AS1^Oex^ lentivirus at a concentration equivalent to 29.7 ng of p24/ml for every 9.6 cm^2^ of cells, in post-fusion medium containing polybrene (5 μg/ml). After 18 to 20 hours, the viral medium was discarded, and the myotubes were washed once with PBS before being cultured in standard post-fusion medium. Terminal experiments were carried out 48 and/or 96 hours later.

### RNA-FISH combined with immunocytochemistry

RNA-FISH combined with immunocytochemistry assays for the detection of TMEM9B-AS1 and desmin were performed using the ViewRNA Cell Plus Assay Kit (Invitrogen) according to the manufacturer’s instructions. Briefly, primary human skeletal muscle cells were seeded and differentiated on laminin (Sigma-Aldrich)–coated four-well Nunc Lab-Tek II Chamber Slides (Thermo Fisher Scientific). At day 8 of differentiation, human myotubes were washed with PBS containing ribonuclease (RNase) inhibitor and fixed with fixation/permeabilization solution that are provided in the kit for 30 min at room temperature. Anti-desmin antibody (1:500), secondary goat anti-rabbit antibody, Alexa Fluor 594 (1:1000), and a probe against TMEM9B-AS1 (VA4-3102848-VCP, Thermo Fisher Scientific) were used for detection. First, antibody staining was performed by incubating the cells for 1 hour with the primary anti-desmin antibody, followed by washes with 1× PBS with RNase inhibitor and 1 hour of incubation with the secondary antibody. The RNA probe against TMEM9B-AS1 was diluted 1:100 and subsequently hybridized for 2 hours at 40°C. After hybridization and amplification steps with preamplifier, amplifier, and linked labeled probe, the probes were detected using Alexa Fluor dyes, according to the manufacturer’s instructions. The cells were mounted using ProLong Gold Antifade Mountant with 4′,6-diamidino-2-phenylindole (DAPI, Invitrogen). Images were obtained using a Leica TCS SP8 confocal microscope (Leica Microsystems) and 63× oil objectives. Images were analyzed and adjusted for brightness and contrast using ImageJ/Fiji software (*56*).

### RNA isolation and cDNA synthesis

For RNA extraction and purification, the Quick-RNA kit from Zymo Research was used. The cells used in the experiments were washed with PBS (pH 7.4), lysed, scraped into the lysis buffer provided in the kit, and snap frozen with liquid nitrogen. To isolate RNA, the samples were allowed to thaw to room temperature before proceeding with kit purification and isolation. RNA concentration and purity were assessed using spectrophotometry (NanoDrop, Thermo Fisher Scientific). For cDNA synthesis, the High-Capacity cDNA Reverse Transcription kit (Thermo Fisher Scientific) was used according to the manufacturer’s instructions, with 1 μg of purified total RNA as starting material. In short, a combination of random primers, deoxynucleotide triphosphate, RT buffer, and the MultiScribe Reverse Transcriptase enzyme were added to the RNA samples. Reverse transcription was performed, commencing with a 10-min step at 25°C, followed by a 120-min incubation at 37°C to facilitate cDNA polymerization. Subsequently, the samples were subjected to a 5-min heat treatment at 85°C to halt the reaction.

### qPCR experiments

Gene expression was determined by real-time qPCR using the Viia7 system (Thermo Fisher Scientific). Results were then analyzed using relative gene expression and calculated using the ΔΔCt according to housekeeping/reference genes with stable expression levels. Unless otherwise specified, the geometric mean of two or more housekeeping genes was used for analysis. qPCR was performed using TaqMan Fast Universal PCR Master Mix (Applied Biosystems) or Fast SYBR Green Master Mix (Applied Biosystems), and primers or predesigned probes (Thermo Fisher Scientific) are listed in table S2. When performing qPCR experiments with TaqMan probes, mRNA expression levels were normalized to housekeeping genes also detected using TaqMan technology. Similarly, for qPCR experiments conducted with SYBR Green, gene expression was normalized to reference genes measured with SYBR primers.

### Western blot experiments

Human skeletal muscle cells were deprived of serum for 4 hours, and, subsequently, they were exposed to insulin for a 10-min period for the stimulated conditions. The cells were then lysed and homogenized using a lysis buffer consisting of 137 mM NaCl, 2.7 mM KCl, 1 mM MgCl_2_, 0.5 mM Na_3_VO_4_, 1% (v/v) Triton X-100, 10% (v/v) glycerol, 20 mM tris (pH 7.8), 1 mM EDTA, with the addition of 1 mM phenylmethylsulfonyl fluoride (PMSF), 1% (v/v) Protease Inhibitor Cocktail Set 1 (Merck Millipore), and 1× PhosSTOP (Roche) before use. These lysates were gently rotated at 4°C for 30 min before subjecting to centrifugation at 12,000*g* at 4°C for 15 min. The protein concentration was measured using the Pierce BCA (bicinchoninic acid) Protein Assay Kit (Thermo Fisher Scientific), and equal quantities of protein were mixed with Laemmli buffer or 5× Pierce Lane Marker Reducing Sample Buffer (Thermo Fisher Scientific). The samples were boiled for 5 to 10 min and then separated using SDS–polyacrylamide gel electrophoresis with 4 to 12% Criterion XT bis-tris gels (Bio-Rad). The proteins were transferred to polyvinylidene difluoride membranes (Merck Millipore), and Ponceau S (Sigma-Aldrich) staining was performed. Following this, the membranes were blocked in 10% milk in TBS-T (10 mM tris-HCl, 100 mM NaCl, and 0.02% Tween 20) (pH 7.6) for 1 hour at room temperature and subsequently incubated at 4°C overnight with 1:1000 diluted primary antibodies (listed in table S2). The membranes were then washed with TBS-T, exposed to suitable secondary antibodies in 5% milk containing TBS-T with a dilution of 1:10000 for 1 hour at room temperature, and then washed again with TBS-T. Chemiluminescence detection was conducted using Amersham ECL or Amersham ECL select Western blotting Detection Reagent (GE Healthcare). Protein content was quantified through densitometry (using QuantityOne software from Bio-Rad or ImageJ). The complete scanned blots are available in figs. S10 to S12.

### SUnSET assay

The protocol for SUnSET assay is adapted from Schmidt *et al.* (*57*). Human myotubes were washed once with PBS and starved in low-glucose (1 g/liter) DMEM for 4 to 5 hours. Puromycin (1 μM final) was then added to the media and incubated for 30 min. Before the harvest, the cells were washed with ice-cold PBS and scraped into lysis buffer consisting of 137 mM NaCl, 2.7 mM KCl, 1 mM MgCl2, 1% (v/v) Triton X-100, 10% (v/v) glycerol, 20 mM tris (pH 7.8), 1 mM EDTA, 1 mM PMSF, 1% (v/v) Protease Inhibitor Cocktail Set 1 (Merck Millipore), and 1× PhosSTOP (Roche). These lysates were gently rotated at 4°C for 30 min before being subjected to centrifugation at 12,000*g* at 4°C for 15 min. The supernatant was collected and stored at −80°C until sample preparation for immunoblot analysis.

### In vitro transcription and 3′end desthiobiotinylation

gBlocks gene fragments (Integrated DNA Technologies) were ordered for TMEM9B-AS1 and MYC-CRD that contains the sequence of T7 RNA polymerase promoter region for in vitro transcription. They were prepared and kept according to the manufacturer’s instructions.

T7 RNA polymerase (New England Biolabs) and components for the standard RNA synthesis were mixed and incubated at 37°C for 3 to 4 hours. Immediately after this incubation, Turbo DNase (0.1 U/μl ; Invitrogen) was added, and the mixture was incubated for an additional 15 min at 37°C. Next, 25 μl of RNase-free water was added to the mixture and transcribed RNA was eluted using the RNA Clean & Concentrator kit (Zymo Research). The samples were assessed for their length and concentration by Bioanalyzer (Agilent) and Nanodrop (Thermo Fisher Scientific).

Pierce RNA 3′ End Desthiobiotinylation Kit (Thermo Fisher Scientific) was used for the biotinylation of the transcribed RNA for the pull-down experiments. Approximately 30 pmol of RNA was used for desthiobiotinylation. To relax the secondary structure, RNA was heated for 3 to 5 min at 85°C in the presence of 25% dimethyl sulfoxide (provided in the kit) and cooled down rapidly on ice. Ligation reactions for desthiobiotinylation were prepared according to the manufacturer’s instructions and were incubated overnight at 16°C. The next day, desthiobitinylated RNA was extracted using chloroform:isoamyl alcohol and the RNA pellet was resuspended in nuclease-free water. The final concentration of desthiobiotinlylated RNA was measured using Nanodrop. Thirty ρmol of desthiobiotinylated control or test RNA was used for purifications.

### RNA pull-down experiments

Fifteen-centimeter dishes were seeded with primary human skeletal muscle cells, and differentiation was done in these plates. Cells were harvested at day 9 after differentiation for the pull-down experiments. For the siRNA silencing experiments, cell media were changed to post-fusion at day 5. Double transfections were performed at day 6 and day 8 of differentiation. The cells were transfected with control stealth RNA interference (RNAi) (control #1, Medium GC, Invitrogen) or with TMEM9B-AS1 stealth RNAi (HSS162366, Thermo Fisher Scientific; https://www.thermofisher.com/order/genome-database/details/sirna/HSS162366?CID=&ICID=&subtype=sirna_stealth). Then, 10 days after the start of differentiation, the cells were scraped and harvested in 300 μl of IP lysis buffer [25 mM tris-HCl (pH 7.4), 150 mM NaCl, 1% NP-40, 1 mM EDTA, and 5% glycerol], supplemented with Protease Inhibitor Cocktail Set 1 (Merck Millipore) and RNase inhibitors (Applied Biosystems). The cell lysates were gently rotated at 4°C for 30 min before being subjected to centrifugation at 12,000*g* at 4°C for 15 min, and the supernatant was kept on ice for the pull-down experiments. Thirty microliters of the lysate was used per pull-down condition. Proteins were pulled down either on TMEM9B-AS1–bound streptavidin beads or control RNA (PolyA)–bound streptavidin beads. Pierce Magnetic RNA-Protein Pull-Down Kit was used for the experiments (Thermo Fisher Scientific). For the rest of the protocol, the manufacturer’s instructions and recommendations were followed.

### Sample preparation for mass spectrometry

Protein eluents (~50 μl) were supplemented with 1 μl of 1 M ammonium bicarbonate (AmBic) and digested with 2 μl of sequencing grade trypsin (0.05 μg/μl; Promega) in 100 mM AmBic, incubating at 37°C overnight with shaking at 450 rpm on a thermal heater. The digestion was stopped with 1 μl of cc. formic acid (FA), and then the samples were transferred to a vial and dried in a vacuum concentrator (Eppendorf).

### Liquid chromatography–tandem mass spectrometry data acquisition

Peptides were reconstituted in 7 μl of solvent A (0.1% FA in water), and 5 μl was injected on a 50-cm-long EASY-Spray C18 column (Thermo Fisher Scientific) connected to an UltiMate 3000 nanoUPLC system (Thermo Fisher Scientific) using a 60-min-long gradient: 4 to 26% of solvent B (98% acetonitrile and 0.1% FA) in 60 min, 26 to 95% in 5 min, and 95% of solvent B for 5 min at a flow rate of 300 nl/min. Mass spectra were acquired on a Q Exactive HF hybrid quadrupole Orbitrap mass spectrometer (Thermo Fisher Scientific) ranging from mass/charge ratio (*m*/*z*) 375 to 1500 at a resolution of *R* = 120,000 (at *m*/*z* 200) targeting 5 × 106 ions for maximum injection time of 100 ms, followed by data-dependent higher-energy collisional dissociation fragmentations of precursor ions with a charge state 2+ to 7+, using 30-s dynamic exclusion. The tandem mass spectra of the top 17 precursor ions were acquired with a resolution of *R* = 30,000, targeting 2 × 105 ions for maximum injection time of 54 ms, setting quadrupole isolation width to 1.4 Th and normalized collision energy to 28%.

### Protein identification

Acquired raw data files were analyzed using Proteome Discoverer v2.4 (Thermo Fisher Scientific) with Mascot Server v5.1 (MatrixScience, UK) against human protein database (SwissProt). A maximum of two missed cleavage sites were allowed for full tryptic digestion, while setting the precursor and the fragment ion mass tolerance to 10 ppm and 0.02 Da, respectively. Carbamidomethylation of cysteine was specified as a fixed modification. Oxidation on methionine and deamidation of asparagine and glutamine were set as dynamic modifications.

### Selection of the protein interactors of TMEM9B-AS1

Proteomics data of control and TMEM9B-AS1 pulldowns were compared. First, proteins that only appeared in TMEM9B-AS1 pulldown were identified for each experiment/donor. Then, peak intensities for the peptides were used to identify proteins having abundance at least 10-fold in TMEM9B-AS1 pulldown in comparison to the control pulldown for each experiment/donor. Combined lists of protein interactors were created for each donor (three donors in total), and these lists were compared with each other and overlapping proteins were determined as specific interactors as shown in Venn diagrams in Fig. 3E and in table S1.

### Glucose oxidation assay

Differentiated human myotubes were serum-starved for 2 hours in low-glucose, 5.5 mM DMEM (Gibco). Then, the medium was changed to the one supplemented with D-[U-^14^C] glucose in the presence or absence of 1 μM carbonyl cyanide *p*-trifluoromethoxyphenylhydrazone (FCCP, Sigma-Aldrich). An empty cup was put in each well, and plates were sealed and incubated for 4 hours at 37°C in 0% CO_2_ incubator. After the incubation, the medium was acidified (1:8 vol 2 M HCl) and the liberated ^14^CO_2_ was collected for an hour in a well containing 2 M NaOH. The quantification for the liberated ^14^CO_2_ was determined by scintillation counting. The cells were harvested in 400 μl 0.5 M NaOH, and pH was neutralized by the addition of 100 μl of 2 M HCl. Protein content was determined by BCA Protein Assay Kit (Thermo Fisher Scientific).

### Fatty acid (palmitate) oxidation assay

Differentiated human myotubes were serum-starved for 2 hours in low-glucose DMEM, 5.5 mM (Gibco) and then were incubated for 6 hours in low-glucose DMEM supplemented with 25 μM of palmitate, including 0.078 μM [9,10-^3^H(N)] palmitate (PerkinElmer) and 0.04% bovine serum albumin (BSA) and in the presence or absence of 1 μM FCCP (Sigma-Aldrich). Supernatant was collected and incubated with 800 μl of 10% activated charcoal in 20 mM tris-HCl buffer (pH 7.5) for 30 min. Samples were centrifuged at 13,000*g* for 15 min. Two hundred microliters of the supernatant was counted in a liquid scintillation counter. Protein content of the cells was measured using the BCA Assay Kit (Thermo Fisher Scientific), and the counts were normalized to the protein content of the cells.

### Glucose uptake assay

Differentiated human myotubes were serum-starved for 4 hours in low-glucose, 5.5 mM (Gibco) and then were incubated for 60 min, at 37°C in the presence or absence of insulin (100 nmol/liter). Then, the cells were treated with glucose- and serum-free DMEM (Gibco) with the addition of 2-[1,2-3H]deoxy-d-glucose (Moravek) and unlabeled 2-deoxy-d-glucose (10 μmol/liter) for 15 min. Upon incubation, the cells were washed three times with ice-cold PBS and were lysed in 1 ml of 0.03% SDS. Five hundred microliters of this lysate was transferred to the scintillation vials and counted in a 1414 WinSpectral Liquid scintillation counter. Protein content of the cells was measured using the BCA Assay Kit (Thermo Fisher Scientific), and the counts were normalized to the protein content of the cells.

### mRNA stability assay

Upon siRNA transfections, the cells were treated either with Actinomycin D (Sigma-Aldrich) (4-nM final concentration) or vehicle. The samples were collected at basal, 10, 20, and 30 min after the treatment in lysis buffer for RNA isolations and were immediately frozen in liquid nitrogen. EZNA total RNA kit (Omega) was used for RNA isolation, and High-Capacity cDNA Reverse Transcription kit (Thermo Fisher Scientific) was used for cDNA synthesis by following the manufacturer’s instructions. MYC mRNA levels were measured by qPCR, and data were normalized to the expression levels of the housekeeping gene, TBP.

### Immunocytochemistry

Primary human skeletal muscle cells were seeded and differentiated on four-well Nunc Lab-Tek II Chamber Slides (Thermo Fisher Scientific) coated with laminin (Sigma-Aldrich) according to the manufacturer’s instructions. Double transfection was performed for siRNA silencing of TMEM9B-AS1 and IGF2BP1 at day 6 and day 8 after the differentiation has started. At day 10, the cells were fixed for 10 min with 4% paraformaldehyde and quenched with 0.1 M glycine for 10 min at room temperature. The cells were washed two times with PBS and were permeabilized for 3 min with 0.1% Triton X-100 in PBS (pH 7.4). After two washes with PBS, the samples were blocked for 30 min in 1% BSA in PBS and incubated with primary antibody for desmin [1:500 diluted in PBS (pH 7.4)] overnight at 4°C. Upon two washes with 0.025% Tween 20 in PBS, secondary antibody incubation was performed for 90 min at room temperature using Alexa Fluor 546 (1:1000 diluted in PBS). After two washes with 0.025% Tween 20 in PBS (pH 7.4), the cells were mounted using ProLong Gold Antifade Mountant with DAPI (Invitrogen). Images were obtained using a Leica Stellaris 5 LIA confocal microscope (Leica Microsystems) and 20× dry objectives. A random region was selected on the slides, and nine pictures were taken including and surrounding that area. The images were stitched together to have the final field of view for comparisons. The images were analyzed and adjusted for brightness and contrast using ImageJ/Fiji software (*56*).

### STRING, KEGG pathway, and GO term analysis for protein interactors

STRING database versions 11.5 and 12.0 were used for the protein-protein interaction networks and KEGG Pathway and GO term enrichment analysis (*58*). Default settings were used for the predictions with the usage of confidence as meaning of network edges. The line thickness represents the strength of the interaction.

### GTEx expression overview

Bulk tissue gene expression information for TMEM9B-AS1was achieved from the GTEx portal (*59*). Data were extracted for the sex-specific expression of TMEM9B-AS1 in different metabolic tissues.

### FANTOM 5 expression overview

The expression level of IGF2BP1 in different human tissues was derived from the FANTOM5 dataset (*60*) and is represented on Human Protein Atlas version 23.0. (*61*).

### Gene expression profiles for TMEM9B-AS1 from Gene Expression Omnibus

GEO2R with the default settings was used to analyze the datasets GSE111006 and GSE165630. Specific gene expression profiles for TMEM9B-AS1 were dissected, and sample values were extracted. Statistical analyses were conducted using Welch *t* test.

### Statistical analysis

GraphPad Prism 10 software (GraphPad Software Inc., CA) was used for the statistical analyses. Normal distribution of the data was tested with Shapiro-Wilk test. For the datasets that were normally distributed, differences between two groups were assessed using paired two-tailed *t* test or one-sample *t* test. For the datasets that are not following normal distribution, Welch *t* test or Wilcoxon test were applied to assess differences between two groups. Differences between more than two groups were assessed by using one-way analysis of variance (ANOVA) followed by Dunnett’s test or Tukey’s test for post hoc pair-wise comparisons. To analyze the effect of two independent variables and calculate the interaction effect we used two-way ANOVA and Tukey, Sidak, or uncorrected Fisher’s least significant difference post hoc test for multiple comparisons. Individual data points are shown in the plots and graphs, and the variation is presented as SD together with the mean of the datasets. *P* values for significances are as follows: *P < 0.05; **P ≤ 0.01; ***P ≤ 0.001; ****P ≤ 0.0001.

## Supplementary Materials

### The PDF file includes:

Figs. S1 to S12 Legend for table S1 Table S2 References
