# Supplementary material for: Down-regulation of human-specific lncRNA TMEM9B-AS1 in skeletal muscle of people with type 2 diabetes affects ribosomal biogenesis
Source: Sci Adv. 2025 Jul 9;11(28):eads4371. doi: 10.1126/sciadv.ads4371 (PMC12239959; doi:10.1126/sciadv.ads4371)
Supplement: Supplementary file 1 — Figs. S1 to S12 Legend for table S1 Table S2 References [file sciadv.ads4371_sm.pdf]

Science Advances
AAAS

Supplementary Materials for

Down-regulation of human-specific lncRNA TMEM9B-AS1 in skeletal muscle of people with type 2 diabetes affects ribosomal biogenesis

Ilke Sen *et al.*

Corresponding author: Ilke Sen, ilke.sen@ki.se; Anna Krook, anna.krook@ki.se

Sci. Adv. **11**, eads4371 (2025)
DOI: 10.1126/sciadv.ads4371

The PDF file includes:

- Figs. S1 to S12
- Legend for table S1
- Table S2
- References

Other Supplementary Material for this manuscript includes the following:

- Table S1

Figure S1. TMEM9B-AS1 levels do not change in human skeletal muscle cells upon Metformin and Simvastatin treatment. (A) qPCR (TaqMan assay) results showing the expression levels of TMEM9B-AS1 upon treatment of human myotubes with Metformin at three different doses; low (5uM), medium (200uM) and high (2mM) and Simvastatin (5uM). Data are normalized to the housekeeping/reference genes B2M and TBP (n=6). For the statistical analysis, a one-way ANOVA test with post-hoc Dunnett's test for comparisons to control was performed. Experiments were performed using biological replicates.

A)

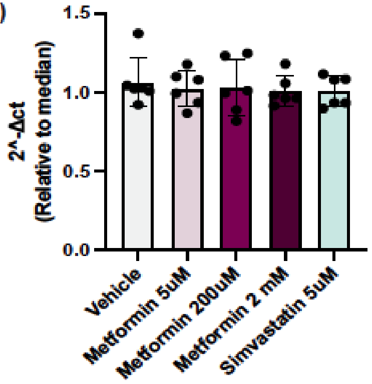

Figure S2. TMEM9B-AS1 regulates the expression of essential proteins that are required for muscle structure and contractility but not differentiation. (A) Western blot results of Desmin (n=7), **(B)** MYH7 (MHC I) (n=5), **(C)** MYH1/2 (MHC II) (n=6), **(D)** Myogenin (n=7) in control and TMEM9B-AS1 siRNA treated human myotubes. Band area ratios were calculated by the normalization of the band intensity of the protein of interest to a selected ponceau band. For the statistical analysis, a two-tailed, paired t-test was performed; * $p<0.05$, ** $p<0.01$. **(E)** qPCR (TaqMan assay) results showing the expression levels of muscle differentiation markers PAX7, MYOD1 and MYOG upon TMEM9B-AS1 silencing, (n=7). Experiments were performed using biological replicates.

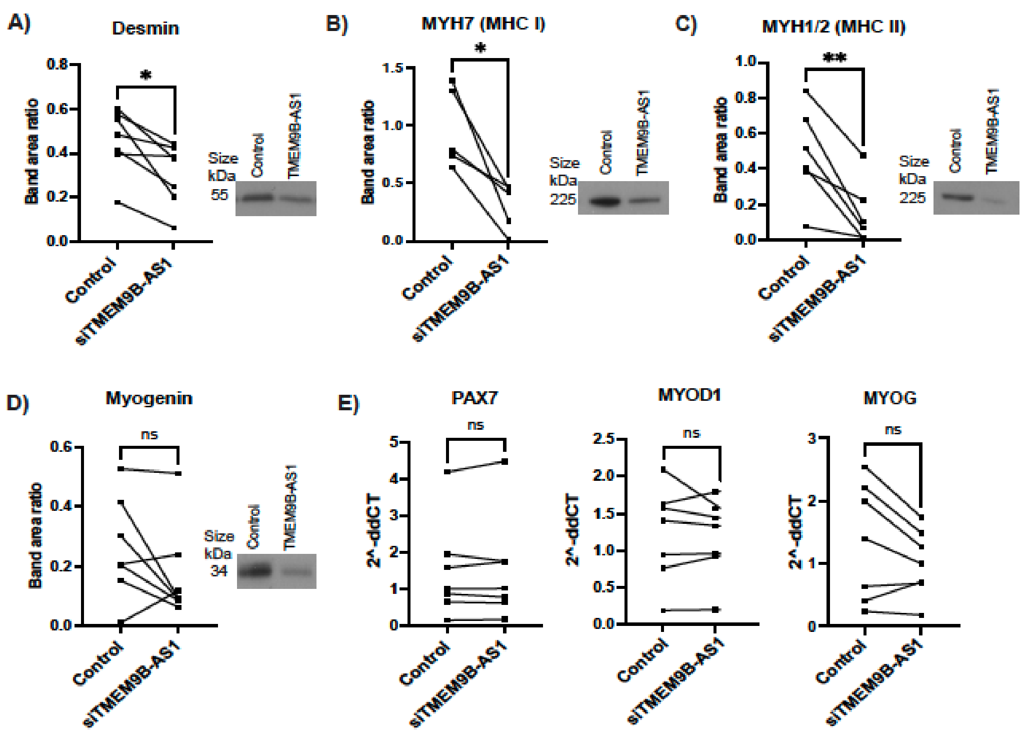

Figure S3. TMEM9B-AS1 does not regulate glucose or lipid metabolism in human myotubes. (A) Glucose oxidation (n=4), **(B)** Palmitic acid (lipid) oxidation (n=4) and **(C)** Glucose uptake (n=6) assay results in human myotubes. Measurements were done at baseline or upon a stimulation with FCCP (for oxidation assays) and insulin (for glucose uptake). Results were normalized to total protein content of the myotubes. For the statistical analysis, a RM two-way ANOVA test was performed. Experiments were performed using biological replicates.

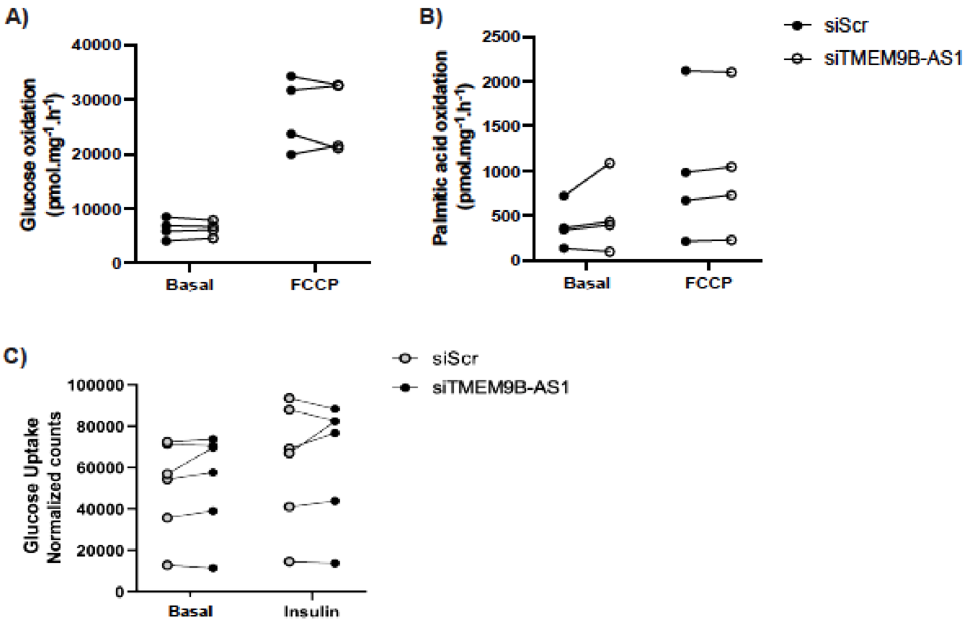

Figure S4. TMEM9B-AS1 overexpression increases translation capacity and TMEM9B-AS1 silencing does not change the phosphorylation level of mTOR nor 4E-BP1 in human myotubes. (A) qPCR (TaqMan assay) results showing the expression level of TMEM9B-AS1 upon overexpression of TMEM9B-AS1 48 and 96 hours after the transduction. B2M, HPRT1 and PPIA were used as housekeeping genes (n=6). For the statistical analysis, RM two-way ANOVA was performed followed by uncorrected Fisher's LSD test for multiple comparisons; * $p<0.05$. **(B)** Total RNA levels of human myotubes upon overexpression of TMEM9B-AS1 48 and 96 hours after the transduction (n=6). For the statistical analysis, two-way RM ANOVA was performed followed by Sidak post-hoc test for multiple comparisons; * $p<0.05$. **(C)** Western blot results of puromycin following a SUnSET assay (n=5), For the statistical analysis, a two-tailed, paired t-test was performed; ** $p<0.01$. Phosphorylation level of **(D)** mTOR (n=6), **(E)** 4E-BP1 (n=7) upon TMEM9B-AS1 silencing. Phosphorylation level of **(F)** S6 protein on Ser 235/236 (n=6), **(G)** S6 protein on Ser 240/244 (n=6) upon overexpression of TMEM9B-AS1. For the statistical analysis, a one-sample t-test was performed; * $p<0.05$. Band intensities were normalized to a Ponceau band. Phosphorylation levels were normalized to total protein levels. Experiments were performed using biological replicates.

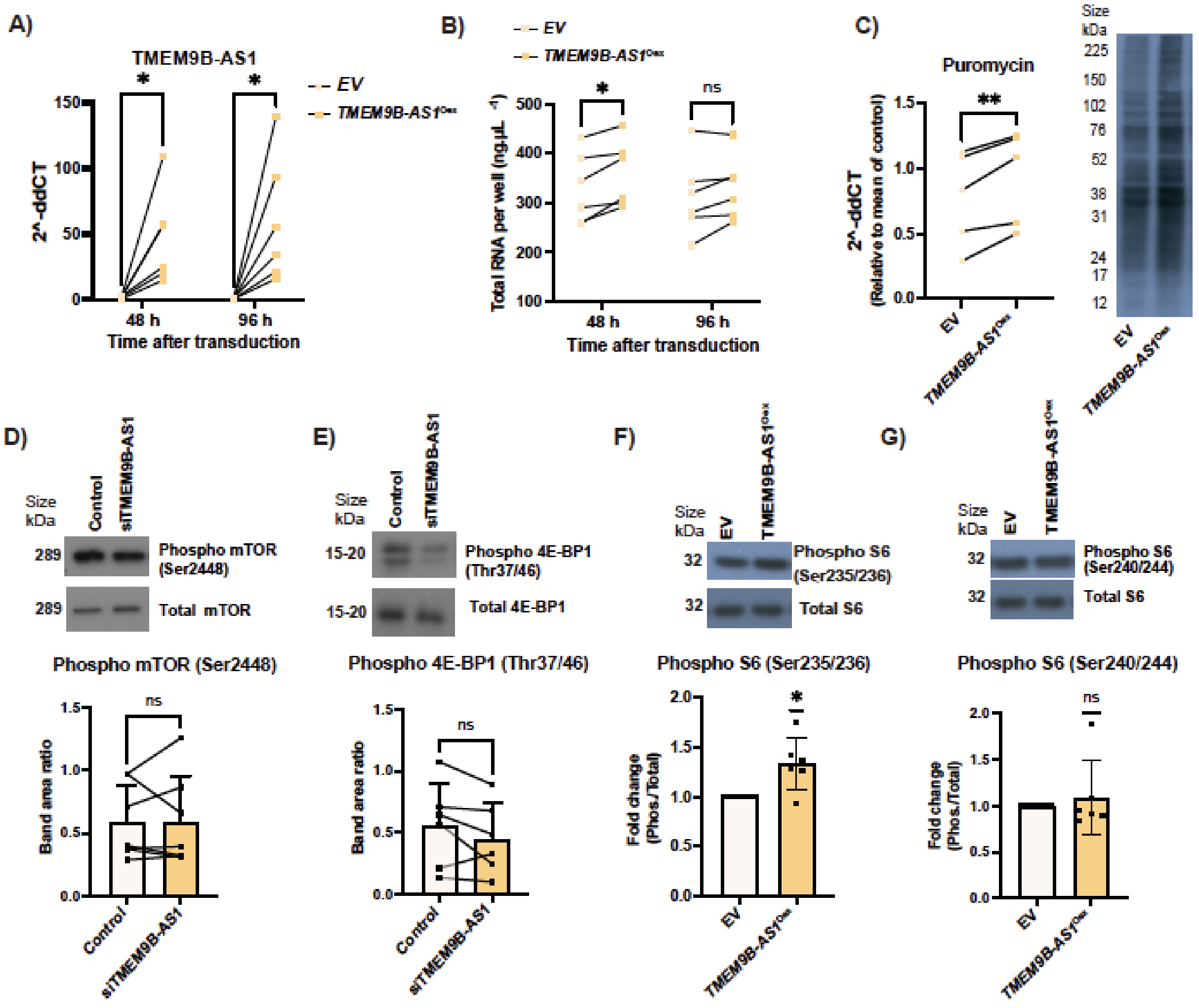

Figure S5. TMEM9B-AS1 overexpression did not affect the abundance of ribosomal genes/RNAs or ribosomal subunit proteins. qPCR (Syber green) results showing expression levels of **(A)** ribosomal RNAs, 28S and 18S **(B)** ribosomal genes, RPS6, RPL11 and RPLP0 **(C)** ribosomal RNA, 7SL_1 upon TMEM9B-AS1 overexpression (48- and 96-hours post-transduction). B2M and GUSB were used as housekeeping genes (n=6). For the statistical analysis, RM two-way ANOVA was performed followed by uncorrected Fisher's LSD test for multiple comparisons. **(D)** Western blot results of ribosomal subunit proteins RPS6, RPLP0, RPL11 and RPL22 in human myotubes upon overexpression of TMEM9B-AS1 (96 hours post-transduction), n=6. Band intensities were normalized to a Ponceau band. For the statistical analysis, a one-sample t-test was performed. Experiments were performed using biological replicates.

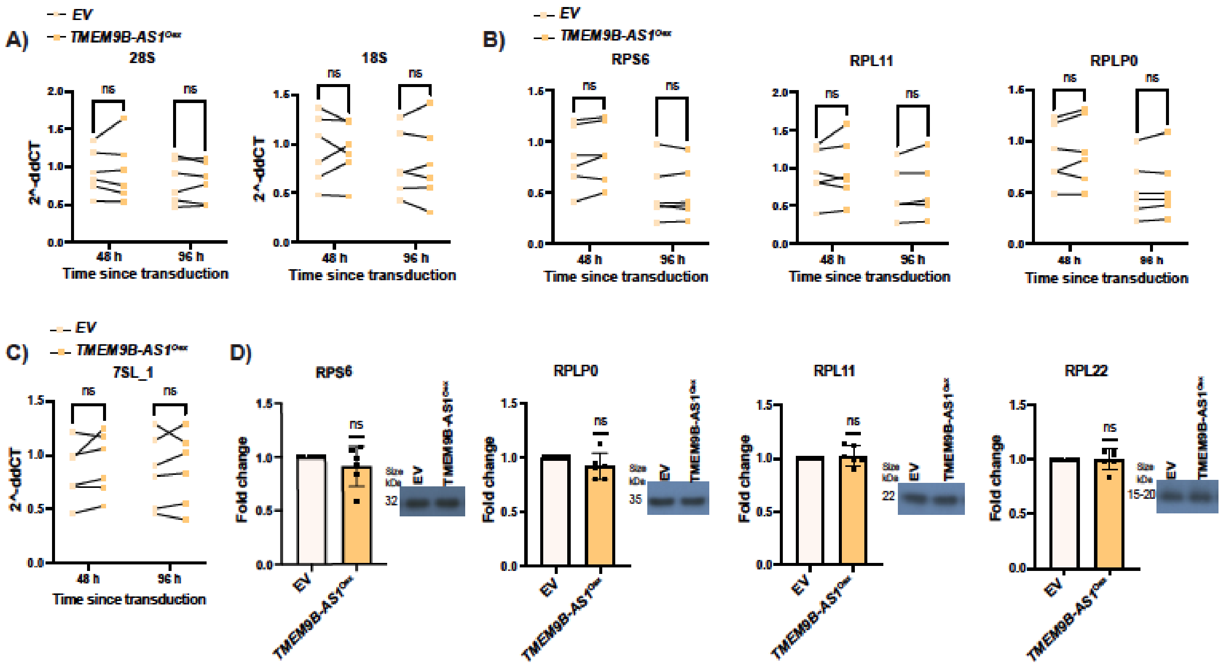

Figure S6. IGF2BP1 protein level is regulated by TMEM9B-AS1 and MYC translation is not affected by the loss of TMEM9B-AS1 nor IGF2BPs. Western blot results showing the protein levels of **(A)** IGF2BP1(n=8) **(B)** IGF2BP2 (n=8) **(C)** MYC (n=6) upon TMEM9B-AS1 silencing in human myotubes. **(D)** Western blot results for MYC protein levels upon IGF2BP1 and IGF2BP2 siRNA silencing (n=6). Band area ratios were calculated by the normalization of the band intensity of the protein of interest to a selected ponceau band. For the statistical analysis, a two-tailed, paired t-test was performed; * p<0.05. Experiments were performed using biological replicates.

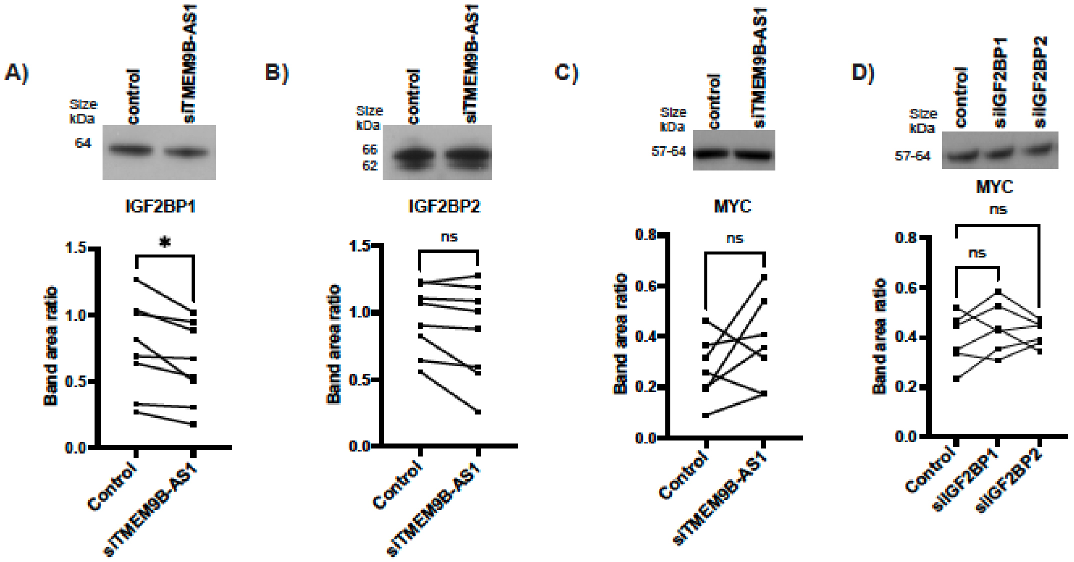

Figure S7. IGF2BP1 is expressed in human skeletal muscle cells/fibers and its expression level is the highest in human myotubes compared to the rodent skeletal muscle cells and tissues. Single tissue expression pattern of **(A)** MYC, **(B)** TMEM9B-AS1, and **(C)** IGF2BP1 on GTEX database (*33*). Arrows are indicating the expression level of TMEM9B-AS1, IGF2BP1 and IGF2BP2 in myocytes and satellite cells in skeletal muscle tissue. **(D)** Expression level of MYC, TMEM9B-AS1 and IGF2BP1 in skeletal muscle fibers and satellite stem cells on the single-cell data platform, CZ CELLxGENE Discover (*34*). **(E)** Comparison of the expression level of IGF2BP1 mRNA in human and rodent skeletal muscle cells and tissues.

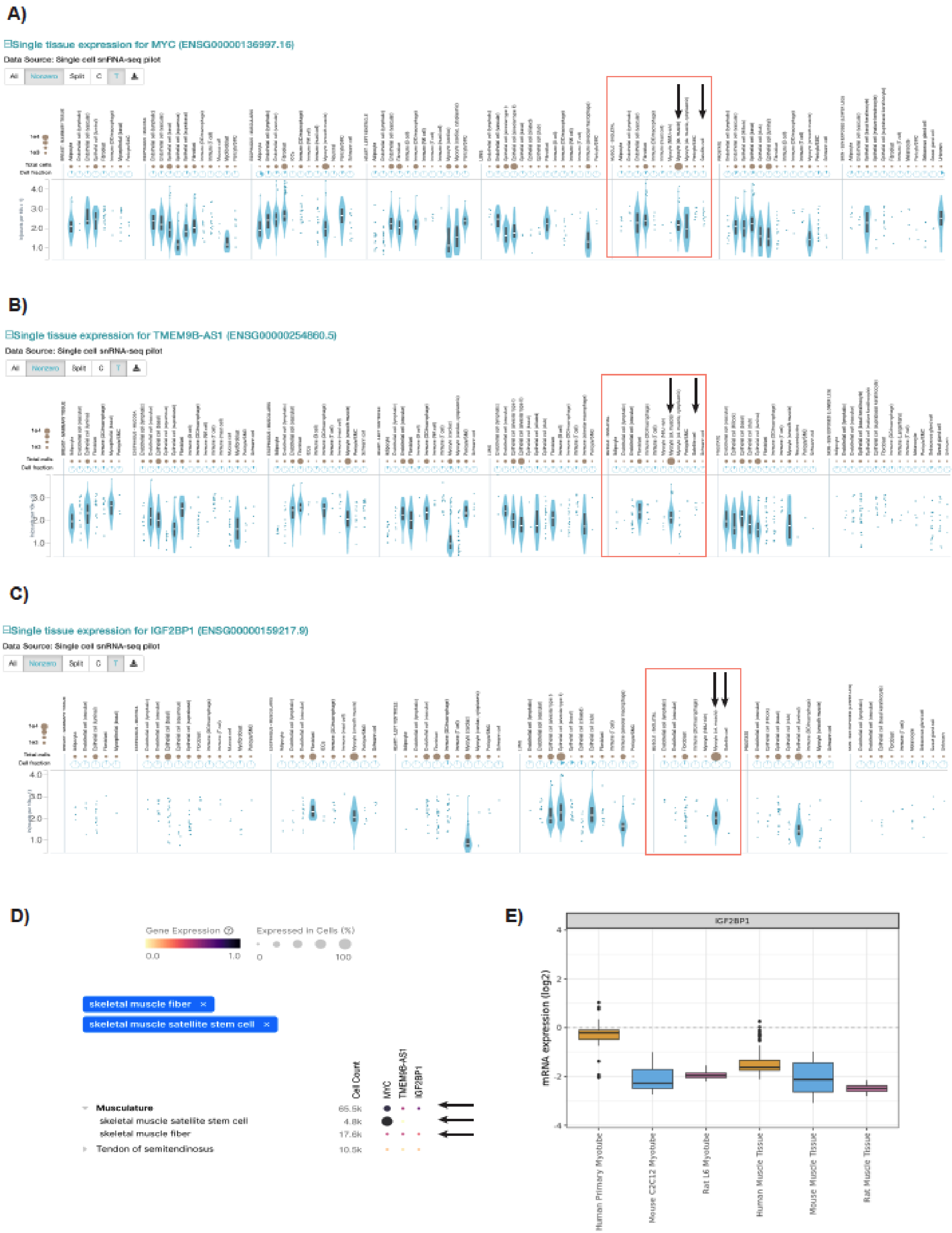
A)
Single tissue expression for MYC (ENSG00000136997.16)
Data Source: Single cell snRNA-seq pilot
All
Nonzero
Split
B)
Single tissue expression for TMEM9B-AS1 (ENSG00000254860.5)
Data Source: Single cell snRNA-seq pilot
C)
Single tissue expression for IGF2BP1 (ENSG00000159217.9)
Data Source: Single cell snRNA-seq pilot
D)
Gene Expression
0.0
1.0
Expressed in Cells (%)
0
100
skeletal muscle fiber
skeletal muscle satellite stem cell
Cell Count
MYC
TMEM9B-AS1
IGF2BP1
Musculature
65.5k
skeletal muscle satellite stem cell
4.8k
skeletal muscle fiber
17.6k
Tendon of semitendinosus
10.5k
E)
IGF2BP1
mRNA expression (log2)
Human Primary Myotube
Mouse C2C12 Myotube
Rat L6 Myotube
Human Muscle Tissue
Mouse Muscle Tissue
Rat Muscle Tissue

Figure S8. TMEM9B-AS1 levels do not change in human skeletal muscle of young individuals upon resistance training. (A) qPCR (TaqMan assay) results of TMEM9B-AS1 in skeletal muscle of young individuals before and after a resistance training intervention (n=26). TBP and B2M were used as housekeeping genes. For the statistical analysis, a two-tailed, paired t-test was performed. Experiments were performed using biological replicates.

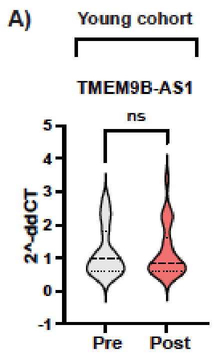

Figure S9. Pairwise statistical analysis of the data presented in Figure 3. qPCR (Syber green) results showing expression levels of **(A)** ribosomal RNAs, 28S and 18S (n=5) **(B)** ribosomal genes, RPS6, RPL11 and RPLP0 (n=6). **(C)** ribosomal RNA, 7SL_1 (n=6) upon TMEM9B-AS1 silencing. TBP and GUSB were used as housekeeping genes. For the statistical analysis, paired two-tailed t-test was performed; * p<0.05, ** p<0.01. **(D)** Western blot results of ribosomal subunit proteins RPL in human myotubes upon silencing of TMEM9B-AS1 (n=7). For the statistical analysis, paired two-tailed t-test was performed; * p<0.05.

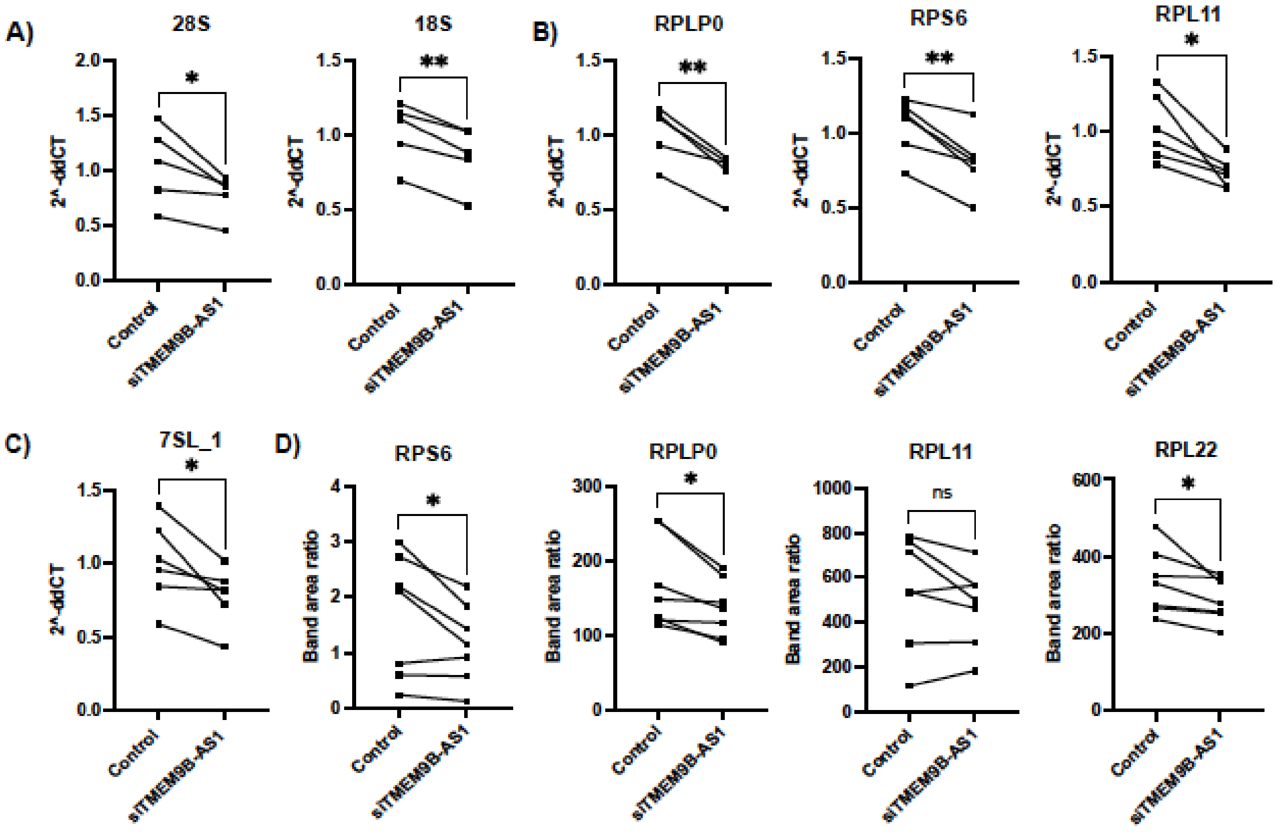

Figure S10. All membranes part I. Uncut membranes and ponceau staining with the information about the sample sets/replicates for **(A)** Puromycin western blot experiment in Fig. 1F, **(B)** Uncut membranes for sample set 1 and 2 for phosphorylated and total mTOR, 4EBP1 (fig. S4, D and E), S6 protein (for Ser235/236 in Fig. 2G), **(C)** Ponceau staining for sample set 1 and 2 for Phosphorylated and total mTOR, 4EBP1 (fig. S4, C and D), S6 protein (for Ser235/236 in Fig. 2G). **(D)** Uncut membranes for the. sample sets 3-6 for phosphorylated and total mTOR, 4EBP1 (fig. S4, D and E), S6 protein (for Ser235/236 in Fig. 2G), **(E)** Ponceau staining for sample the sets 3-6 phosphorylated and total mTOR, 4EBP1 (fig. S4, D and E), and S6 protein (for Ser235/236 in Fig. 2G). **(F)** Uncut membranes for the. sample sets 7 for phosphorylated and total mTOR, 4EBP1 (fig. S4, D and E), and S6 protein (for Ser235/236 in Fig. 2G), **(G)** Ponceau staining for sample the set 1-7 for phosphorylated and total mTOR, 4EBP1 (fig. S4, D and E), and S6 protein (for Ser235/236 in Fig. 2G). **(H)** Uncut membranes for the sample sets 1-7 for phosphorylated and total S6 protein (for Ser240/244 in Fig. 2H), p70 S6K (Fig. 2I), p90 S6K, RSK (Fig. 2J) and ERK1/2 (Fig. 2K) **(I)** Ponceau staining for the sample sets 1-7 for phosphorylated and total S6 protein (for Ser240/244 in Fig. 2H), p70 S6K (Fig. 2I), p90 S6K, RSK (Fig. 2J) and ERK1/2 (Fig. 2K). **(J)** Uncut membranes for the RNA pulldown experiment in Fig. 4F. **(K)** Ponceau staining for the RNA pulldown experiments in Fig. 4F.

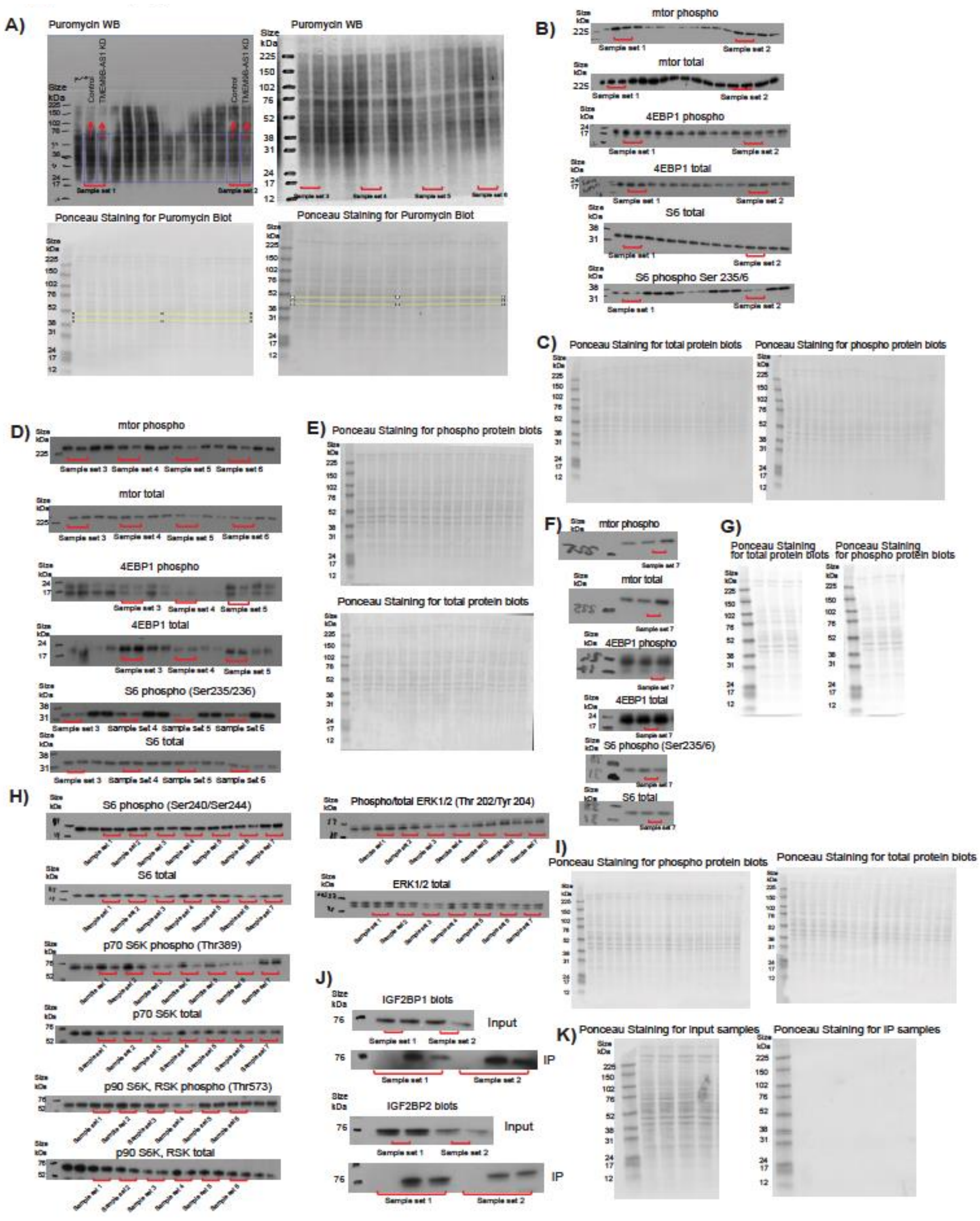
A)
Puromycin WB
Control
TMEM9B-AS1 KD
Size kDa
225
150
102
76
52
38
31
24
17
12
Sample set 1
Sample set 2
Sample set 3
Sample set 4
Sample set 5
Sample set 6
Ponceau Staining for Puromycin Blot
B)
mtor phospho
mtor total
4EBP1 phospho
4EBP1 total
S6 total
S6 phospho Ser 235/6
C)
Ponceau Staining for total protein blots
Ponceau Staining for phospho protein blots
D)
mtor phospho
mtor total
4EBP1 phospho
4EBP1 total
S6 phospho (Ser235/236)
S6 total
E)
Ponceau Staining for phospho protein blots
Ponceau Staining for total protein blots
F)
mtor phospho
mtor total
4EBP1 phospho
4EBP1 total
S6 phospho (Ser235/6)
S6 total
Sample set 7
G)
Ponceau Staining for total protein blots
Ponceau Staining for phospho protein blots
H)
S6 phospho (Ser240/Ser244)
S6 total
p70 S6K phospho (Thr389)
p70 S6K total
p90 S6K, RSK phospho (Thr573)
p90 S6K, RSK total
Phospho/total ERK1/2 (Thr 202/Tyr 204)
ERK1/2 total
I)
Ponceau Staining for phospho protein blots
Ponceau Staining for total protein blots
J)
IGF2BP1 blots
Input
IP
IGF2BP2 blots
K)
Ponceau Staining for input samples
Ponceau Staining for IP samples

Figure S11. All membranes part II. Uncut membranes with the information about the sample sets/replicates for Western blot experiments of **(A)** Total ubiquitinylated proteins (Fig. 2C), **(B)** Atrogin-1/MAFbx (Fig. 2D), **(C)** PAN proteasome 20S alpha (Fig. 2E), **(D)** Desmin (fig. S2A), **(E)** Myogenin (fig. S2D), **(F)** MYH1/2 (MyHC IIX/IIA) (fig. S2C), **(G)** MYH7 (MyHC I) (fig. S2B), **(H)** MYC (fig. S6C), **(I)** MYC (fig. S6D), **(J)** IGF2BP1 (fig. S6A), and **(K)** IGF2BP2 (fig. S6B). Ponceau staining for **(L)** Total ubiquitinylated proteins (Fig. 2C) and PAN proteasome 20S alpha (Fig. 2E), **(M)** Atrogin-1/MAFbx (Fig. 2D), **(N)** Desmin (fig. S2A), Myogenin (fig. S2D), and MYH1/2 (MHC II) (fig. S2C), **(O)** MYH7 (MHC I) (fig. S2B), **(P)** MYC (fig. S6C), **(Q)** MYC (fig. S6D), **(R)** IGF2BP1 (fig. S6A). and **(S)** IGF2BP2 (fig. S6B).

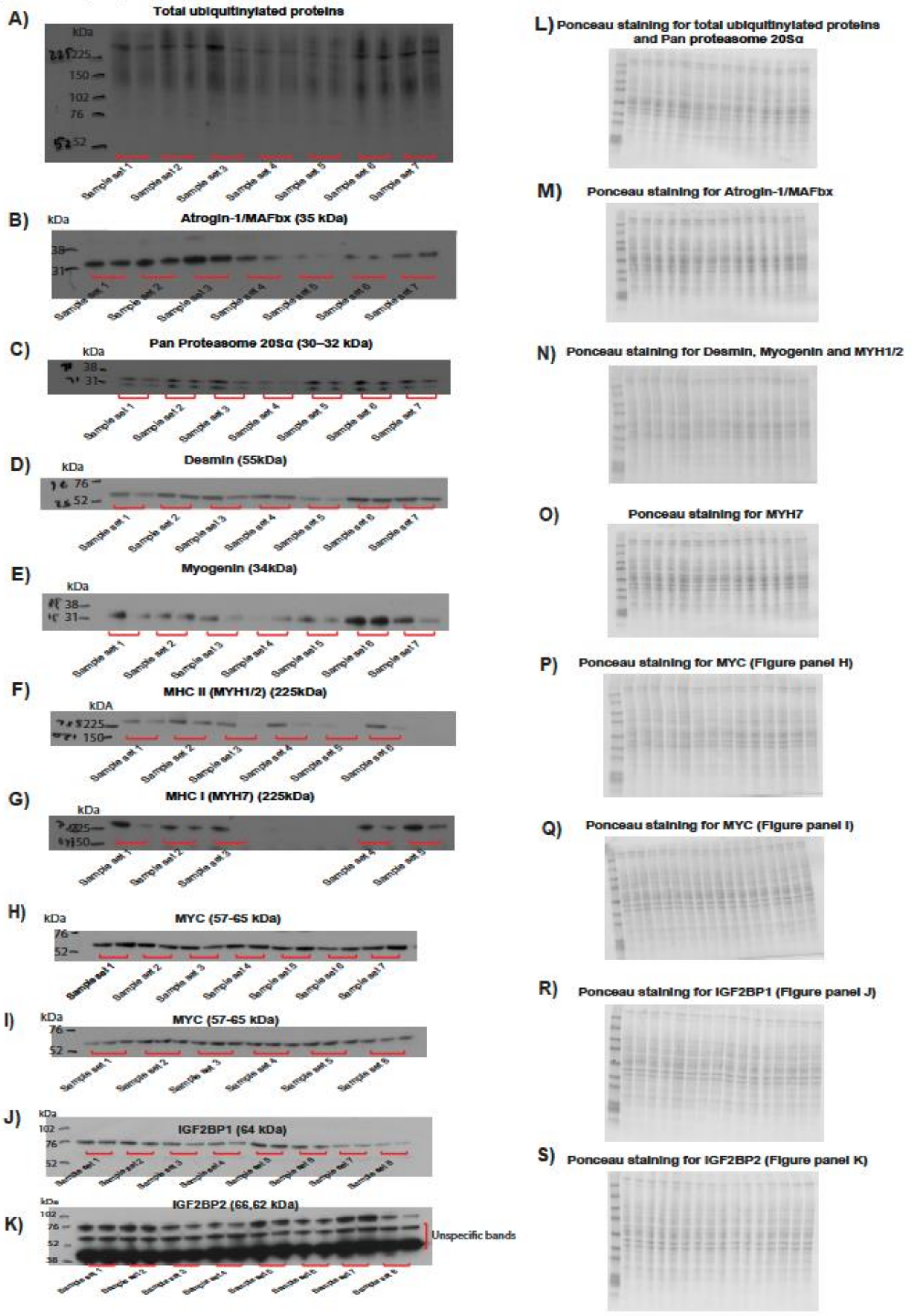
A) Total ubiquitinylated proteins
kDa
225
150
102
76
52
Sample set 1
Sample set 2
Sample set 3
Sample set 4
Sample set 5
Sample set 6
Sample set 7
B) Atrogin-1/MAFbx (35 kDa)
kDa
38
31
C) Pan Proteasome 20Sα (30–32 kDa)
kDa
38
31
D) Desmin (55kDa)
kDa
76
52
E) Myogenin (34kDa)
kDa
38
31
F) MHC II (MYH1/2) (225kDa)
kDA
225
150
G) MHC I (MYH7) (225kDa)
kDa
H) MYC (57-65 kDa)
kDa
76
52
I) MYC (57-65 kDa)
kDa
76
52
J) IGF2BP1 (64 kDa)
kDa
102
76
52
Sample set 8
K) IGF2BP2 (66,62 kDa)
kDa
102
76
52
38
Unspecific bands
L) Ponceau staining for total ubiquitinylated proteins and Pan proteasome 20Sα
M) Ponceau staining for Atrogin-1/MAFbx
N) Ponceau staining for Desmin, Myogenin and MYH1/2
O) Ponceau staining for MYH7
P) Ponceau staining for MYC (Figure panel H)
Q) Ponceau staining for MYC (Figure panel I)
R) Ponceau staining for IGF2BP1 (Figure panel J)
S) Ponceau staining for IGF2BP2 (Figure panel K)

Fig. S12. All membranes part III. Ponceau staining results for **(A)** RPLP0 and RPL22 (Fig. 3E) Uncut membranes with the information about the sample sets/replicates for Western blot experiments for **(B)** RPLP0 (Fig. 3E) and **(C)** RPL22 (Fig. 3E). **(D)** Ponceau staining results for RPL11 (Fig. 3E). **(E)** Uncut membranes with the information about the sample sets/replicates for Western blot experiments for RPL11 (Fig. 3E). Uncut membranes with the information about the sample sets/replicates for Western blot experiments for **(F)** Puromycin (fig. S4C), **(G)** RPL11 and RPLP0 (fig. S5D). **(H)** Ponceau staining results for Puromycin (fig. S4C, figure panel f), RPL11 and RPLP0 (fig. S5D, figure panel g). **(I)** Uncut membranes with information about sample sets/replicates for Western blot experiments for RPL22 and RPS6 (fig. S5D). **(J)** Ponceau staining for RPL22 and RPS6 (fig. S5D, figure panel i). Uncut membranes with the information about the sample sets/replicates for Western blot experiments for **(K)** S6 protein on Ser 235/236 (fig. S4F), **(L)** S6 protein on Ser 240/244 (fig. S4G). Ponceau staining for **(M)** S6 protein on Ser 235/236 (fig. S4F, figure panel K), **(N)** S6 protein on Ser 240/244 (fig. S4G, figure panel L).

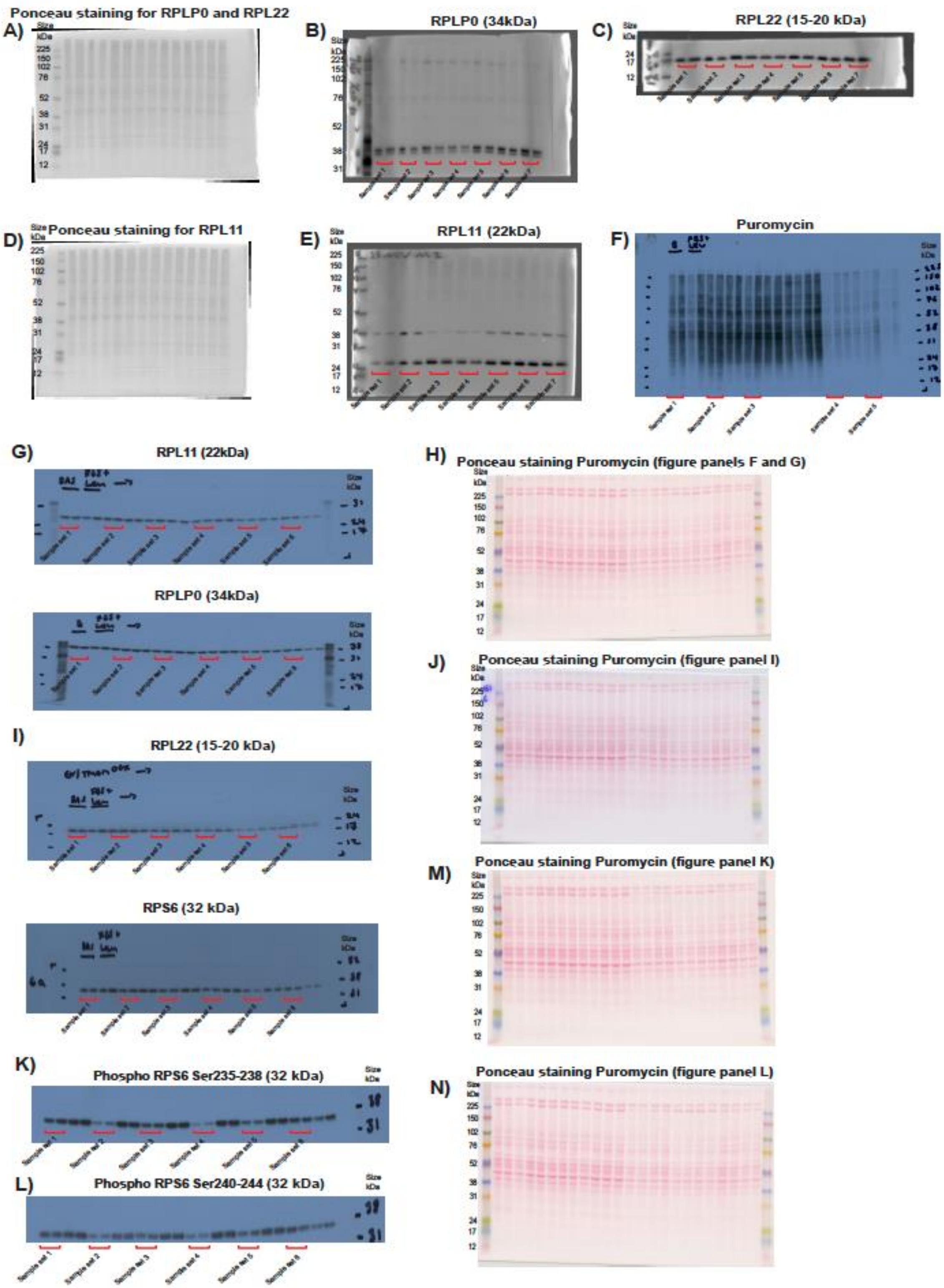

Ponceau staining for RPLP0 and RPL22
A)
B)
RPLP0 (34kDa)
C)
RPL22 (15-20 kDa)
D)
Ponceau staining for RPL11
E)
RPL11 (22kDa)
F)
Puromycin
G)
RPL11 (22kDa)
RPLP0 (34kDa)
H)
Ponceau staining Puromycin (figure panels F and G)
I)
RPL22 (15-20 kDa)
RPS6 (32 kDa)
J)
Ponceau staining Puromycin (figure panel I)
K)
Phospho RPS6 Ser235-238 (32 kDa)
L)
Phospho RPS6 Ser240-244 (32 kDa)
M)
Ponceau staining Puromycin (figure panel K)
N)
Ponceau staining Puromycin (figure panel L)

Table S1. MS results after RNA pulldown experiments and protein interactor lists of TMEM9B-AS1

Table S2. Antibodies, primers and pre-designed TaqMan probes for qPCR experiments

Supplemetary Table 2

18S-mature-f	GATGGTAGTCGCCGTGCC	Kwon et al., 2014 (*63*)
18S-mature-r	GCCTGCTGCCTTCCTTGG	Kwon et al., 2014 (*63*)
28S-mature-f	GTGACGCGCATGAATGGA	Kwon et al., 2014 (*63*)
28S-mature-r	TGTGGTTTCGCTGGATAGTAGGT	Kwon et al 2014 (*63*)
RPS6_F	TCTTGACCCATGGCCGTGTC	Fumagalli et al., 2009 (*64*)
RPS6_R	GCGGCGAGGCACTGTAGTAT	Fumagalli et al., 2009 (*64*)
RPL11_F	TCCACTGCACAGTTCGAGGG	Fumagalli et al., 2009 (*64*)
RPL11_R	AAACCTGGCCTACCCAGCAC	Fumagalli et al., 2009 (*64*)
7SL_F	ATCGGGTGTCCGCACTAAGTT	Tucker et al., 2019 (*65*)
7SL_R	CAGCACGGGAGTTTTGACCT	Tucker et al., 2019 (*65*)
TBP_F	AGTTCTGGGATTGTACCGCA	
TBP_R	TATATTCGGCGTTTCGGGCA	
GUSB_F	GCAGATGTGTGACCGCTATG	
GUSB_R	TGAGCGATCACCATCTTCAAG	
TMEM9B-AS1	TaqMan probe Hs00937602_m1	Thermo Fisher Scientific
MYC	TaqMan probe Hs00153408_m1	Thermo Fisher Scientific
IGF2BP1	TaqMan probe Hs00198023_m1	Thermo Fisher Scientific
IGF2BP2	TaqMan probe Hs01118009_m1	Thermo Fisher Scientific
B2M	TaqMan probe Hs00187842_m1	Thermo Fisher Scientific
HPRT	TaqMan probe Hs02800695_m1	Thermo Fisher Scientific
TBP	TaqMan probe cat. no. 4326322E	Thermo Fisher Scientific
PPIA	TaqMan probe Hs04194521_s1	Thermo Fisher Scientific
PAX7	TaqMan probe Hs00242962_m1	Thermo Fisher Scientific
MYOD1	TaqMan probe Hs00159528_m1	Thermo Fisher Scientific
MYOG	TaqMan probe Hs01072232_m1	Thermo Fisher Scientific

Antibodies:

- DESMIN antibody Rabbit polyclonal, #15200, Abcam
- Alexa Fluor 594 goat, anti-rabbit IgG antibody, Invitrogen
- Anti-Puromycin Antibody, clone 12D10, MABE343, Merck, Sigma-Aldrich
- Phospho-mTOR (Ser2448) Antibody #2971, rabbit, Cell Signaling
- mTOR (7C10) Rabbit mAb #2983, Cell Signaling
- Phospho-4E-BP1 (Thr37/46) (236B4) Rabbit mAb, #2855, Cell Signaling
- 4E-BP1 (53H11) Rabbit mAb #9644, Cell Signaling
- Phospho-S6 Ribosomal Protein (Ser235/236) Antibody, #2211, Cell Signaling
- S6 Ribosomal Protein (54D2) Mouse mAb #2317, Cell Signaling
- Phospho-S6 Ribosomal Protein (Ser240/244) (D68F8) XP® Rabbit mAb #5364, Cell Signaling
- Phospho-p70 S6 Kinase (Thr389) Antibody #9205, Cell Signaling
- p70 S6 Kinase (49D7) Rabbit mAb #2708, Cell Signaling
- Phospho-p90RSK (Thr573) Antibody #9346, Cell Signaling
- RSK2 Antibody #9340, Cell Signaling
- Phospho-p44/42 MAPK (Erk1/2) (Thr202/Tyr204) Antibody #9101, Cell Signaling
- p44/42 MAPK (Erk1/2) Antibody #9102, Cell Signaling
- IMP1 (D33A2) Rabbit mAb #8482, Cell Signaling
- IMP2 (D4R2F) Rabbit mAb #14672, Cell Signaling
- Anti-Proteasome 20S alpha 1+2+3+5+6+7 antibody [MCP231] Mouse mAb #ab22674, Abcam
- Anti-ubiquitinylated proteins antibody (FK2) Mouse mAb, #04-263, Sigma-Aldrich
- MAFbx antibody (F-9) Mouse mAb, #166806, Santa Cruz
- MHC I (MYH 7) (A4.840) Mouse mAb, #53089, Santa Cruz
- MHC II (MYH1/2) (A4.1025) Mouse mAb, #53088, Santa Cruz
- Myogenin (MYOG) (F5D) Mouse mAb, #12732, Santa Cruz
- c-Myc (D84C12) Rabbit mAb #5605, Cell Signaling
- RPL22 Rabbit polyclonal antibody, # 25002-1-AP, Proteintech
- RPL11 (D1P5N) Rabbit mAb #18163, Cell Signaling
- RPLP0 Rabbit polyclonal antibody, # 11290-2-AP, Proteintech

REFERENCES AND NOTES

1. T. Derrien, R. Johnson, G. Bussotti, A. Tanzer, S. Djebali, H. Tilgner, G. Guernec, D. Martin, A. Merkel, D. G. Knowles, J. Lagarde, L. Veeravalli, X. Ruan, Y. Ruan, T. Lassmann, P. Carninci, J. B. Brown, L. Lipovich, J. M. Gonzalez, M. Thomas, C. A. Davis, R. Shiekhattar, T. R. Gingeras, T. J. Hubbard, C. Notredame, J. Harrow, R. Guigó, The GENCODE v7 catalog of human long noncoding RNAs: Analysis of their gene structure, evolution, and expression. *Genome Res.* **22**, 1775–1789 (2012).

2. R.-W. Yao, Y. Wang, L.-L. Chen, Cellular functions of long noncoding RNAs. *Nat. Cell Biol.* **21**, 542–551 (2019).

3. T.-N. Zhang, W. Wang, N. Yang, X.-M. Huang, C.-F. Liu, Regulation of glucose and lipid metabolism by long non-coding RNAs: Facts and research progress. *Front. Endocrinol.* **11**, 457 (2020).

4. R. P. Juni, K. C. ’t Hart, R. H. Houtkooper, R. A. Boon, Long noncoding RNAs in cardiometabolic disorders. *FEBS Lett.* **596**, 1367–1387 (2022).

5. R. A. Defronzo, D. Tripathy, Skeletal muscle insulin resistance is the primary defect in type 2 diabetes. *Diabetes Care* **32**, S157–S163 (2009).

6. M. Leenders, L. B. Verdijk, L. van der Hoeven, J. J. Adam, J. van Kranenburg, R. Nilwik, L. J. C. van Loon, Patients with type 2 diabetes show a greater decline in muscle mass, muscle strength, and functional capacity with aging. *J. Am. Med. Dir. Assoc.* **14**, 585–592 (2013).

7. I. J. Neeland, J. Linge, A. L. Birkenfeld, Changes in lean body mass with glucagon-like peptide-1-based therapies and mitigation strategies. *Diabetes. Obes. Metab.* **26**, 16–27 (2024).

8. C. M. Prado, S. M. Phillips, M. C. Gonzalez, S. B. Heymsfield, Muscle matters: The effects of medically induced weight loss on skeletal muscle. *Lancet.* **12**, 785–787 (2024).

9. C. Conte, K. D. Hall, S. Klein, Is weight loss–induced muscle mass loss clinically relevant? *JAMA* **332**, 9–10 (2024).

10. C. Gong, Z. Li, K. Ramanujan, I. Clay, Y. Zhang, S. Lemire-Brachat, D. J. Glass, A long non-coding RNA, LncMyoD, regulates skeletal muscle differentiation by blocking IMP2-mediated mRNA translation. *Dev. Cell* **34**, 181–191 (2015).

11. V. C. Figueiredo, J. J. McCarthy, Regulation of ribosome biogenesis in skeletal muscle hypertrophy. *Phys. Ther.* **34**, 30–42 (2019).

12. V. C. Figueiredo, R. F. D'Souza, D. W. Van Pelt, M. M. Lawrence, N. Zeng, J. F. Markworth, S. D. Poppitt, B. F. Miller, C. J. Mitchell, J. J. McCarthy, E. E. Dupont-Versteegden, D. Cameron-Smith, Ribosome biogenesis and degradation regulate translational capacity during muscle disuse and reloading. *J. Cachexia. Sarcopenia Muscle* **12**, 130–143 (2021).

13. L. Jiao, Y. Liu, X.-Y. Yu, X. Pan, Y. Zhang, J. Tu, Y.-H. Song, Y. Li, Ribosome biogenesis in disease: New players and therapeutic targets. *Signal Transduct. Target. Ther.* **8**, 15 (2023).

14. F. v. Walden, Ribosome biogenesis in skeletal muscle: Coordination of transcription and translation. *J. Appl. Physiol.* **127**, 591–598 (2019).

15. T. Mori, S. Ato, J. R. Knudsen, C. Henriquez-Olguin, Z. Li, K. Wakabayashi, T. Suginohara, K. Higashida, Y. Tamura, K. Nakazato, T. E. Jensen, R. Ogasawara, c-Myc overexpression increases ribosome biogenesis and protein synthesis independent of mTORC1 activation in mouse skeletal muscle. *Am. J. Physiol. Endocrinol. Metab.* **321**, E551–E559 (2021).

16. N. J. Pillon, J. A. B. Smith, P. S. Alm, A. V. Chibalin, J. Alhusen, E. Arner, P. Carninci, T. Fritz, J. Otten, T. Olsson, S. van Doorslaer de ten Ryen, L. Deldicque, K. Caidahl, H. Wallberg-Henriksson, A. Krook, J. R. Zierath, Distinctive exercise-induced inflammatory response and exerkine induction in skeletal muscle of people with type 2 diabetes. *Sci. Adv.* **8**, eabo3192 (2022).

17. B. M. Gabriel, A. Altıntaş, J. A. B. Smith, L. Sardon-Puig, X. Zhang, A. L. Basse, R. C. Laker, H. Gao, Z. Liu, L. Dollet, J. T. Treebak, A. Zorzano, Z. Huo, M. Rydén, J. T. Lanner, K. A. Esser, R. Barrès, N. J. Pillon, A. Krook, J. R. Zierath, Disrupted circadian oscillations in type 2

diabetes are linked to altered rhythmic mitochondrial metabolism in skeletal muscle. *Sci. Adv.* **7**, eabi9654 (2021).

18. C. F. Bentzinger, Y. X. Wang, N. A. Dumont, M. A. Rudnicki, Cellular dynamics in the muscle satellite cell niche. *EMBO Rep.* **14**, 1062–1072 (2013).

19. B. S. Gordon, A. R. Kelleher, S. R. Kimball, Regulation of muscle protein synthesis and the effects of catabolic states. *Int. J. Biochem. Cell Biol.* **45**, 2147–2157 (2013).

20. R. Sartori, V. Romanello, M. Sandri, Mechanisms of muscle atrophy and hypertrophy: Implications in health and disease. *Nat. Commun.* **12**, 330 (2021).

21. I. Sahu, S. M. Mali, P. Sulkshane, C. Xu, A. Rozenberg, R. Morag, M. P. Sahoo, S. K. Singh, Z. Ding, Y. Wang, S. Day, Y. Cong, O. Kleifeld, A. Brik, M. H. Glickman, The 20S as a stand-alone proteasome in cells can degrade the ubiquitin tag. *Nat. Commun.* **12**, 6173 (2021).

22. S. C. Bodine, E. Latres, S. Baumhueter, V. K. M. Lai, L. Nunez, B. A. Clarke, W. T. Poueymirou, F. J. Panaro, E. Na, K. Dharmarajan, Z.-Q. Pan, D. M. Valenzuela, T. M. Dechiara, T. N. Stitt, G. D. Yancopoulos, D. J. Glass, Identification of ubiquitin ligases required for skeletal muscle atrophy. *Science* **294**, 1704–1708 (2001).

23. X. M. Ma, J. Blenis, Molecular mechanisms of mTOR-mediated translational control. *Nat. Rev. Mol. Cell Biol.* **10**, 307–318 (2009).

24. J. Van Riggelen, A. Yetil, D. W. Felsher, MYC as a regulator of ribosome biogenesis and protein synthesis. *Nat. Rev. Cancer* **10**, 301–309 (2010).

25. P. P. Roux, D. Shahbazian, H. Vu, M. K. Holz, M. S. Cohen, J. Taunton, N. Sonenberg, J. Blenis, RAS/ERK signaling promotes site-specific ribosomal protein S6 phosphorylation via RSK and stimulates cap-dependent translation. *J. Biol. Chem.* **282**, 14056–14064 (2007).

26. M.-A. Bjornsti, P. J. Houghton, The tor pathway: A target for cancer therapy. *Nat. Rev. Cancer* **4**, 335–348 (2004).

27. C. Chauvin, V. Koka, A. Nouschi, V. Mieulet, C. Hoareau-Aveilla, A. Dreazen, N. Cagnard, W. Carpentier, T. Kiss, O. Meyuhas, M. Pende, Ribosomal protein S6 kinase activity controls the ribosome biogenesis transcriptional program. *Oncogene* **33**, 474–483 (2014).

28. I. Lemm, J. Ross, Regulation of c-myc mRNA decay by translational pausing in a coding region instability determinant. *Mol. Cell. Biol.* **22**, 3959–3969 (2002).

29. C. Zeng, T. Fukunaga, M. Hamada, Identification and analysis of ribosome-associated lncRNAs using ribosome profiling data. *BMC Genomics* **19**, 414 (2018).

30. J.-H. Li, S. Liu, H. Zhou, L.-H. Qu, J.-H. Yang, starBase v2.0: Decoding miRNA-ceRNA, miRNA-ncRNA and protein–RNA interaction networks from large-scale CLIP-Seq data. *Nucleic Acids Res.* **42**, D92–D97 (2014).

31. N. Degrauwe, M.-L. Suvà, M. Janiszewska, N. Riggi, I. Stamenkovic, IMPs: An RNA-binding protein family that provides a link between stem cell maintenance in normal development and cancer. *Genes Dev.* **30**, 2459–2474 (2016).

32. D. Weidensdorfer, N. Stöhr, A. Baude, M. Lederer, M. Köhn, A. Schierhorn, S. Buchmeier, E. Wahle, S. Hüttelmaier, Control of c-myc mRNA stability by IGF2BP1-associated cytoplasmic RNPs. *RNA* **15**, 104–115 (2009).

33. The GTEX Consortium, The GTEx Consortium atlas of genetic regulatory effects across human tissues. *Science* **369**, 1318–1330 (2020).

34. CZI Cell Science Program, S. Abdulla, B. Aevermann, P. Assis, S. Badajoz, S. M. Bell, E. Bezzi, B. Cakir, J. Chaffer, S. Chambers, T. Chi, J. Chien, L. Dorman, P. Garcia-Nieto, N. Gloria, M. Hastie, D. Hegeman, J. Hilton, T. Huang, A. Infeld, A.-M. Istrate, I. Jelic, K. Katsuya, Y. J. Kim, K. Liang, M. Lin, M. Lombardo, B. Marshall, B. Martin, F. McDade, C. Megill, N. Patel, A. Predeus, B. Raymor, B. Robatmili, D. Rogers, E. Rutherford, D. Sadgat, A. Shin, C. Small, T. Smith, P. Sridharan, A. Tarashansky, N. Tavares, H. Thomas, A. Tolopko, M. Urisko, J. Yan, G. Yeretssian, J. Zamanian, A. Mani, J. Cool, A. Carr, CZ CELLxGENE discover: A

single-cell data platform for scalable exploration, analysis and modeling of aggregated data. *Nucleic Acids Res.* **53**, D886–D900 (2025).

35. A. Lambrianidou, E. Sereti, K. Soupsana, C. Komini, K. Dimas, T. Trangas, mTORC2 deploys the mRNA binding protein IGF2BP1 to regulate c-MYC expression and promote cell survival. *Cell. Signal.* **80**, 109912 (2021).

36. C. Dani, J. M. Blanchard, M. Piechaczyk, S. El Sabouty, L. Marty, P. Jeanteur, Extreme instability of myc mRNA in normal and transformed human cells. *Proc. Natl. Acad. Sci. U.S.A.* **81**, 7046–7050 (1984).

37. M. Linial, N. Gunderson, M. Groudine, Enhanced transcription of c-*myc* in bursal lymphoma cells requires continuous protein synthesis. *Science* **230**, 1126–1132 (1985).

38. S. W. Park, B. H. Goodpaster, E. S. Strotmeyer, L. H. Kuller, R. Broudeau, C. Kammerer, N. De Rekeneire, T. B. Harris, A. V. Schwartz, F. A. Tylavsky, Y.-W. Cho, A. B. Newman, Accelerated loss of skeletal muscle strength in older adults with type 2 diabetes. *Diabetes Care* **30**, 1507–1512 (2007).

39. S. W. Park, B. H. Goodpaster, J. S. Lee, L. H. Kuller, R. Boudreau, N. De Rekeneire, T. B. Harris, S. Kritchevsky, F. A. Tylavsky, M. Nevitt, Y.-W. Cho, A. B. Newman, Excessive loss of skeletal muscle mass in older adults with type 2 diabetes. *Diabetes Care* **32**, 1993–1997 (2009).

40. Y. Wen, A. P. Alimov, J. J. McCarthy, Ribosome biogenesis is necessary for skeletal muscle hypertrophy. *Exerc. Sport Sci. Rev.* **44**, 110–115 (2016).

41. K. Baar, K. Esser, Phosphorylation of p70S6kcorrelates with increased skeletal muscle mass following resistance exercise. *Am. J. Physiol. Cell Physiol.* **276**, C120–C127 (1999).

42. P. De Sanctis, G. Filardo, P. M. Abruzzo, A. Astolfi, A. Bolotta, V. Indio, A. Di Martino, C. Hofer, H. Kern, S. Löfler, M. Marcacci, M. Marini, S. Zampieri, C. Zucchini, Non-coding RNAs in the transcriptional network that differentiates skeletal muscles of sedentary from long-term endurance- and resistance-trained elderly. *Int. J. Mol. Sci.* **22**, 1539 (2021).

43. D. Hammarström, S. J. Øfsteng, N. B. Jacobsen, K. B. Flobergseter, B. R. Rønnestad, S. Ellefsen, Ribosome accumulation during early phase resistance training in humans. *Acta Physiol.* **235**, e13806 (2022).

44. K. Lian, D. Hammarström, H. Hamarsland, K. S. Mølmen, S. C. Moen, S. Ellefsen, Glucose ingestion before and after resistance training sessions does not augment ribosome biogenesis in healthy moderately trained young adults. *Eur. J. Appl. Physiol.* **124**, 2329–2342 (2024).

45. E. Migliavacca, S. K. H. Tay, H. P. Patel, T. Sonntag, G. Civiletto, C. McFarlane, T. Forrester, S. J. Barton, M. K. Leow, E. Antoun, A. Charpagne, Y. Seng Chong, P. Descombes, L. Feng, P. Francis-Emmanuel, E. S. Garratt, M. P. Giner, C. O. Green, S. Karaz, N. Kothandaraman, J. Marquis, S. Metairon, S. Moco, G. Nelson, S. Ngo, T. Pleasants, F. Raymond, A. A. Sayer, C. Ming Sim, J. Slater-Jefferies, H. E. Syddall, P. Fang Tan, P. Titcombe, C. Vaz, L. D. Westbury, G. Wong, W. Yonghui, C. Cooper, A. Sheppard, K. M. Godfrey, K. A. Lillycrop, N. Karnani, J. N. Feige, Mitochondrial oxidative capacity and NAD+ biosynthesis are reduced in human sarcopenia across ethnicities. *Nat. Commun.* **10**, 5808 (2019).

46. I. Ruvinsky, M. Katz, A. Dreazen, Y. Gielchinsky, A. Saada, N. Freedman, E. Mishani, G. Zimmerman, J. Kasir, O. Meyuhas, Mice deficient in ribosomal protein S6 phosphorylation suffer from muscle weakness that reflects a growth defect and energy deficit. *PLOS ONE* **4**, e5618 (2009).

47. K. Yaniv, J. K. Yisraeli, The involvement of a conserved family of RNA binding proteins in embryonic development and carcinogenesis. *Gene* **287**, 49–54 (2002).

48. H. Weng, F. Huang, Z. Yu, Z. Chen, E. Prince, Y. Kang, K. Zhou, W. Li, J. Hu, C. Fu, T. Aziz, H. Li, J. Li, Y. Yang, L. Han, S. Zhang, Y. Ma, M. Sun, H. Wu, Z. Zhang, M. Wunderlich, S. Robinson, D. Braas, J. T. Hoeve, B. Zhang, G. Marcucci, J. C. Mulloy, K. Zhou, H.-F. Tao, X. Deng, D. Horne, M. Wei, H. Huang, J. Chen, The m6A reader IGF2BP2 regulates glutamine metabolism and represents a therapeutic target in acute myeloid leukemia. *Cancer Cell* **40**, 1566–1582.e10 (2022).

49. N. Dai, The diverse functions of IMP2/IGF2BP2 in metabolism. *Trends Endocrinol. Metab.* **31**, 670–679 (2020).

50. J. Wang, L. Chen, P. Qiang, The role of IGF2BP2, an m6A reader gene, in human metabolic diseases and cancers. *Cancer Cell Int.* **21**, 99 (2021).

51. M. C. Costanzo, M. Von Grotthuss, J. Massung, D. Jang, L. Caulkins, R. Koesterer, C. Gilbert, R. P. Welch, P. Kudtarkar, Q. Hoang, A. P. Boughton, P. Singh, Y. Sun, M. Duby, A. Moriondo, T. Nguyen, P. Smadbeck, B. R. Alexander, M. Brandes, M. Carmichael, P. Dornbos, T. Green, K. C. Huellas-Bruskiewicz, Y. Ji, A. Kluge, A. C. McMahon, J. M. Mercader, O. Ruebenacker, S. Sengupta, D. Spalding, D. Taliun, AMP–T2D Consortium, P. Smith, M. K. Thomas, B. Akolkar, M. J. Brosnan, A. Cherkas, A. Y. Chu, E. B. Fauman, C. S. Fox, T. N. Kamphaus, M. R. Miller, L. Nguyen, A. Parsa, D. F. Reilly, H. Ruetten, D. Wholley, N. A. Zaghloul, G. R. Abecasis, D. Altshuler, T. M. Keane, M. I. McCarthy, K. J. Gaulton, J. C. Florez, M. Boehnke, N. P. Burtt, J. Flannick, The Type 2 Diabetes Knowledge Portal: An open access genetic resource dedicated to type 2 diabetes and related traits. *Cell Metab.* **35**, 695–710.e6 (2023).

52. H. Huang, H. Weng, W. Sun, X. Qin, H. Shi, H. Wu, B. S. Zhao, A. Mesquita, C. Liu, C. L. Yuan, Y.-C. Hu, S. Hüttelmaier, J. R. Skibbe, R. Su, X. Deng, L. Dong, M. Sun, C. Li, S. Nachtergaele, Y. Wang, C. Hu, K. Ferchen, K. D. Greis, X. Jiang, M. Wei, L. Qu, J.-L. Guan, C. He, J. Yang, J. Chen, Recognition of RNA N6-methyladenosine by IGF2BP proteins enhances mRNA stability and translation. *Nat. Cell Biol.* **20**, 285–295 (2018).

53. M. S. Brook, D. J. Wilkinson, W. K. Mitchell, J. N. Lund, B. E. Phillips, N. J. Szewczyk, P. L. Greenhaff, K. Smith, P. J. Atherton, Synchronous deficits in cumulative muscle protein synthesis and ribosomal biogenesis underlie age-related anabolic resistance to exercise in humans. *J. Physiol.* **594**, 7399–7417 (2016).

54. J. Lin, Y. Wen, J. Tang, X. Zhang, H. Zhang, H. Zhu. Human-specific lncRNAs contributed critically to human evolution by distinctly regulating gene expression. *eLife* **12**, RP89001 (2023).

55. L. Al-Khalili, D. Krämer, P. Wretenberg, A. Krook, Human skeletal muscle cell differentiation is associated with changes in myogenic markers and enhanced insulin-mediated MAPK and PKB phosphorylation. *Acta Physiol. Scand.* **180**, 395–403 (2004).

56. J. Schindelin, I. Arganda-Carreras, E. Frise, V. Kaynig, M. Longair, T. Pietzsch, S. Preibisch, C. Rueden, S. Saalfeld, B. Schmid, J.-Y. Tinevez, D. J. White, V. Hartenstein, K. Eliceiri, P. Tomancak, A. Cardona, Fiji: An open-source platform for biological-image analysis. *Nat. Methods* **9**, 676–682 (2012).

57. E. K. Schmidt, G. Clavarino, M. Ceppi, P. Pierre, SUnSET, a nonradioactive method to monitor protein synthesis. *Nat. Methods* **6**, 275–277 (2009).

58. D. Szklarczyk, A. L. Gable, K. C. Nastou, D. Lyon, R. Kirsch, S. Pyysalo, N. T. Doncheva, M. Legeay, T. Fang, P. Bork, L. J. Jensen, C. von Mering, The STRING database in 2021: Customizable protein–protein networks, and functional characterization of user-uploaded gene/measurement sets. *Nucleic Acids Res.* **49**, D605–D612 (2020).

59. J. Lonsdale, J. Thomas, M. Salvatore, R. Phillips, E. Lo, S. Shad, R. Hasz, G. Walters, F. Garcia, N. Young, B. Foster, M. Moser, E. Karasik, B. Gillard, K. Ramsey, S. Sullivan, J. Bridge, H. Magazine, J. Syron, J. Fleming, L. Siminoff, H. Traino, M. Mosavel, L. Barker, S. Jewell, D. Rohrer, D. Maxim, D. Filkins, P. Harbach, E. Cortadillo, B. Berghuis, L. Turner, E. Hudson, K. Feenstra, L. Sobin, J. Robb, P. Branton, G. Korzeniewski, C. Shive, D. Tabor, L. Qi, K. Groch, S. Nampally, S. Buia, A. Zimmerman, A. Smith, R. Burges, K. Robinson, K. Valentino, D. Bradbury, M. Cosentino, N. Diaz-Mayoral, M. Kennedy, T. Engel, P. Williams, K. Erickson, K. Ardlie, W. Winckler, G. Getz, D. Deluca, D. Macarthur, M. Kellis, A. Thomson, T. Young, E. Gelfand, M. Donovan, Y. Meng, G. Grant, D. Mash, Y. Marcus, M. Basile, J. Liu, J. Zhu, Z. Tu, N. J. Cox, D. L. Nicolae, E. R. Gamazon, H. K. Im, A. Konkashbaev, J. Pritchard, M. Stevens, T. Flutre, X. Wen, E. T. Dermitzakis, T. Lappalainen, R. Guigo, J. Monlong, M. Sammeth, D. Koller, A. Battle, S. Mostafavi, M. McCarthy, M. Rivas, J. Maller, I. Rusyn, A. Nobel, F. Wright, A. Shabalin, M. Feolo, N. Sharopova, A. Sturcke, J. Paschal, J. M. Anderson, E. L. Wilder, L. K. Derr, E. D. Green, J. P. Struewing, G. Temple, S. Volpi, J. T. Boyer, E. J. Thomson, M. S. Guyer, C. Ng, A. Abdallah, D. Colantuoni, T. R. Insel, S. E. Koester, A. R.

Little, P. K. Bender, T. Lehner, Y. Yao, C. C. Compton, J. B. Vaught, S. Sawyer, N. C. Lockhart, J. Demchok, H. F. Moore, The genotype-tissue expression (GTEx) project. *Nat. Genet.* **45**, 580–585 (2013).

60. The FANTOM Consortium and the RIKEN PMI and CLST (DGT), A promoter-level mammalian expression atlas. *Nature* **507**, 462–470 (2014).

61. M. Karlsson, C. Zhang, L. Méar, W. Zhong, A. Digre, B. Katona, E. Sjöstedt, L. Butler, J. Odeberg, P. Dusart, F. Edfors, P. Oksvold, K. von Feilitzen, M. Zwahlen, M. Arif, O. Altay, X. Li, M. Ozcan, A. Mardinoglu, L. Fagerberg, J. Mulder, Y. Luo, F. Ponten, M. Uhlén, C. Lindskog, A single–cell type transcriptomics map of human tissues. *Sci. Adv.* **7**, eabh2169 (2021).

62. Y. Perez-Riverol, J. Bai, C. Bandla, D. García-Seisdedos, S. Hewapathirana, S. Kamatchinathan, D. J. Kundu, A. Prakash, A. Frericks-Zipper, M. Eisenacher, M. Walzer, S. Wang, A. Brazma, J. A. Vizcaíno, The PRIDE database resources in 2022: A hub for mass spectrometry-based proteomics evidences. *Nucleic Acids Res.* **50**, D543–D552 (2022).

63. I. Kwon, S. Xiang, M. Kato, L. Wu, P. Theodoropoulos, T. Wang, J. Kim, J. Yun, Y. Xie, S. L. McKnight, Poly-dipeptides encoded by the C9orf72 repeats bind nucleoli, impede RNA biogenesis, and kill cells. *Science* **345**, 1139–1145 (2014).

64. S. Fumagalli, A. Di Cara, A. Neb-Gulati, F. Natt, S. Schwemberger, J. Hall, G. F. Babcock, R. Bernardi, P. P. Pandolfi, G. Thomas, Absence of nucleolar disruption after impairment of 40S ribosome biogenesis reveals an rpL11-translation-dependent mechanism of p53 induction. *Nat. Cell Biol.* **11**, 501–508 (2009).

65. J. M. Tucker, A. M. Schaller, I. Willis, B. A. Glaunsinger, Alteration of the premature tRNA landscape by gammaherpesvirus infection. *mBio* **11**, e02664-20 (2020).
